# STING Suppresses Mitochondrial VDAC2 to Govern RCC Growth Independent of Innate Immunity

**DOI:** 10.1002/advs.202203718

**Published:** 2022-11-29

**Authors:** Zhichuan Zhu, Xin Zhou, Hongwei Du, Erica W. Cloer, Jiaming Zhang, Liu Mei, Ying Wang, Xianming Tan, Austin J. Hepperla, Jeremy M. Simon, Jeanette Gowen Cook, Michael B. Major, Gianpietro Dotti, Pengda Liu

**Affiliations:** ^1^ Lineberger Comprehensive Cancer Center The University of North Carolina at Chapel Hill Chapel Hill NC 27599 USA; ^2^ Department of Biochemistry and Biophysics The University of North Carolina at Chapel Hill Chapel Hill NC 27599 USA; ^3^ Department of Microbiology and Immunology The University of North Carolina at Chapel Hill Chapel Hill NC 27599 USA; ^4^ Department of Oral Medicine Infection and Immunity Harvard School of Dental Medicine Boston MA 02115 USA; ^5^ Department of Biostatistics The University of North Carolina at Chapel Hill Chapel Hill NC 27599 USA; ^6^ Carolina Institute for Developmental Disabilities The University of North Carolina at Chapel Hill Chapel Hill NC 27599 USA; ^7^ UNC Neuroscience Center The University of North Carolina at Chapel Hill Chapel Hill NC 27599 USA; ^8^ Department of Genetics The University of North Carolina at Chapel Hill Chapel Hill NC 27599 USA; ^9^ Department of Cell Biology and Physiology Department of Otolaryngology Washington University in St. Louis St. Louis MO 63130 USA

**Keywords:** 2‐BP, innate immunity‐independent, mitochondrial homeostasis, mTORC1, STING, VDAC2

## Abstract

STING is an innate immune sensor for immune surveillance of viral/bacterial infection and maintenance of an immune‐friendly microenvironment to prevent tumorigenesis. However, if and how STING exerts innate immunity‐independent function remains elusive. Here, the authors report that STING expression is increased in renal cell carcinoma (RCC) patients and governs tumor growth through non‐canonical innate immune signaling involving mitochondrial ROS maintenance and calcium homeostasis. Mitochondrial voltage‐dependent anion channel VDAC2 is identified as a new STING binding partner. STING depletion potentiates VDAC2/GRP75‐mediated MERC (mitochondria‐ER contact) formation to increase mitochondrial ROS/calcium levels, impairs mitochondria function, and suppresses mTORC1/S6K signaling leading to RCC growth retardation. STING interaction with VDAC2 occurs through STING‐C88/C91 palmitoylation and inhibiting STING palmitoyl‐transferases ZDHHCs by 2‐BP significantly impedes RCC cell growth alone or in combination with sorafenib. Together, these studies reveal an innate immunity‐independent function of STING in regulating mitochondrial function and growth in RCC, providing a rationale to target the STING/VDAC2 interaction in treating RCC.

## Graphical abstract

STING is a canonical innate immune sensor triggering type‐I interferon production. Here, an innate immunity‐independent STING function in governing renal cancer cell (RCC) proliferation is reported through regulating mitochondria calcium homeostasis by binding mitochondrial calcium transporter VDAC2, as well as its therapeutic potential in treating RCC.

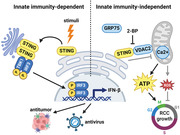

## 1 Introduction

Innate immunity serves as the first line of defense against infectious pathogens in vertebrates to restrain pathogen invasion and activate adaptive immunity. Pathogen‐associated molecular patterns (PAMPs) help distinguish between self‐ and non‐self‐materials and initiate recognition and activation of innate immunity.^[^ ^1^ ^]^ Nucleotides represent a group of important PAMPs to trigger host innate immune responses including both DNA and RNA. Viral and bacterial DNA, as well as damaged genomic and mitochondria DNA, are sensed by the cytosolic DNA sensor cGAS (cyclic GMP‐AMP synthase).^[^ ^2^ ^]^ Binding of DNA to cGAS activates its enzymatic activity by triggering cGAS dimerization and phase transition^[^ ^3^ ^]^ toward synthesizing a special unsymmetric cyclic dinucleotide 2′3′‐cGAMP.^[^ ^4^ ^]^ Subsequently, 2′3′‐cGAMP binds STING (stimulator of interferon response cGAMP interactr 1) on the endoplasmic reticulum (ER) to promote its trafficking to Golgi and other peri‐nuclear membrane‐coated compartments,^[^ ^5^, ^6^ ^]^ where STING recruits TBK1 to phosphorylate IRF3.^[^ ^7^ ^]^ Phosphorylated IRF3 dimerizes and translocates into nuclei in triggering the expression of interferon *β* (IFN*β*).^[^ ^8^ ^]^ This leads to expression of immune modulatory genes^[^ ^9^ ^]^ and activation of adaptive immunity. However, STING can also promote autophagy,^[^ ^10^ ^]^ lysosome‐mediated cell death of myeloid cells,^[^ ^11^ ^]^ and inhibition of viral replication^[^ ^12^ ^]^ via IFN*β* ‐independent mechanisms. In addition, STING also plays a critical role in responding to RNA viruses including influenza A viruses (IAV),^[^ ^13^ ^]^ vesicular stomatitis virus (VSV), dengue virus (DENV)^[^ ^14^ ^]^ and others,^[^ ^15^ ^]^ through directly binding and modulating function of essential RNA sensors RIG‐I and MAVS and other mechanisms.^[^ ^16^, ^17^ ^]^

Other than nucleotide sensing, hyperactivation of STING has been observed and connected with inflammatory diseases from both pathological perspectives (for example, STING‐associated vasculopathy with onset in infancy (SAVI) disease is caused by GOF mutations in STING^[^ ^18^ ^]^) and genetic animal models (for example, systemic lupus erythematosus symptom can be largely rescued in *STING*‐deficient mice^[^ ^19^ ^]^). Moreover, evidence from *Sting^gt/gt^* (*Sting* knockout) mice suggests that activation of the STING signaling creates an immune‐responsive microenvironment that suppresses tumorigenesis,^[^ ^20^ ^]^ and *STING* loss has been observed in colon cancer,^[^ ^21^ ^]^ gastric cancer^[^ ^22^ ^]^ and melanoma^[^ ^23^ ^]^ and restrains IFN‐mediated tissue repair and T cell priming. Activating STING has been shown to improve therapeutic effects of immune‐checkpoint blockades.^[^ ^24^ ^]^ STING also exerts a protective effect in metabolic diseases such as alcoholic liver disease,^[^ ^25^ ^]^ cardiovascular diseases including myocardial infarction,^[^ ^26^ ^]^ aging,^[^ ^27^ ^]^ and others^[^ ^28^ ^]^ through promoting IRF3 phosphorylation and activation of innate immune signaling.

Innate immunity‐independent STING function has also been investigated but is less understood. To this end, although STING activation mediates T cell tumor infiltration, STING was also reported to mediate IFN*β* ‐independent T cell death by inducing ER stress in T cells.^[^ ^29^ ^]^ STING depletion has been shown to promote cancer cell growth via accelerated S‐phase entry and increased chromosome instability in the absence of an immune environment.^[^ ^30^ ^]^ Interestingly, STING activation can mediate mitochondrial damage that leads to renal fibrosis and renal failure.^[^ ^31^, ^32^ ^]^ Although extensive studies demonstrate that STING is involved in controlling tumorigenesis and renal failure, there is no evidence of STING involvement in the development of RCC, especially through a non‐canonical and innate immunity‐independent manner.

## 2 Results

### 2.1 STING Depletion Reduces RCC Cell Growth In Vitro and in Xenograft Mouse Models

**Figure 1:**
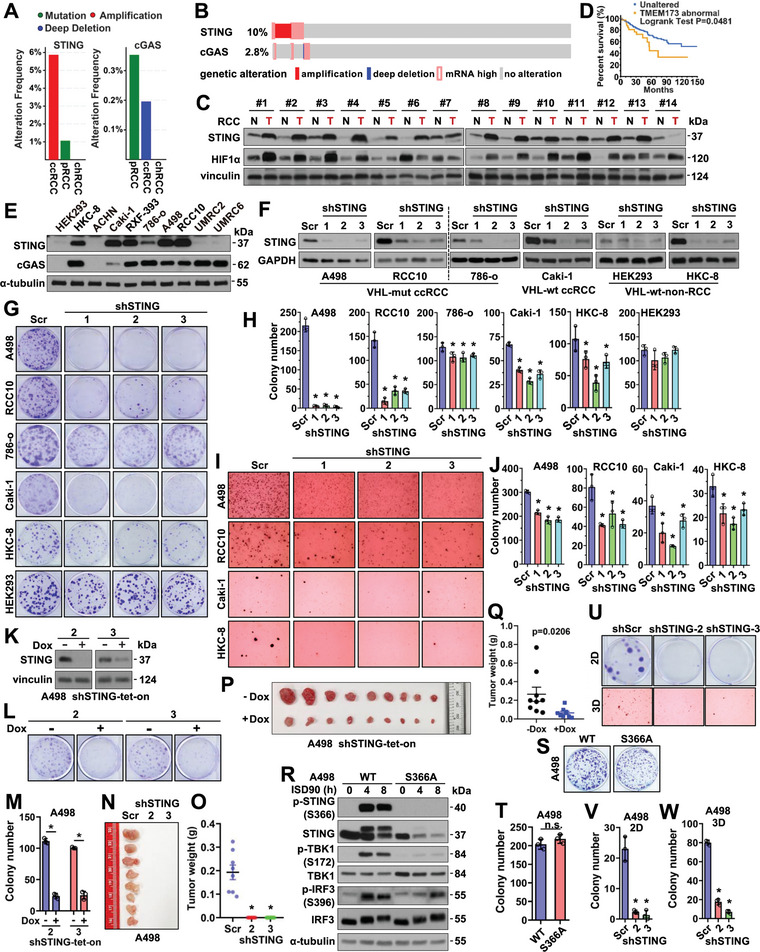
Depletion of STING reduces RCC cell growth independent of innate immunity. A) Genetic alteration frequency (mutated, amplified, and deep deleted cases/total cases) of STING or cGAS in indicated RCC patients from the TCGA PanCancer Atlas database. B) Oncoprint showing genetic alteration of STING and cGAS in ccRCC patients from the TCGA PanCancer Atlas database. C) Immuno‐blot (IB) analyses of tumor lysates from normal‐adjacent tissues (N) or RCC tumors (T). D) A Kaplan‐Meier plot showing increased STING expression is associated with worse patient survival in ccRCC. E) IB analyses of WCL derived from indicated RCC cells. F) IB analyses of control and STING‐depleted RCC cells. G) Representative images for 2D colony formation assays using cells from F. H) Quantification of (G). Error bars were calculated as mean+/‐SD, *n* = 3. *p < 0.05 (one‐way ANOVA test). I, Representative images for 3D soft agar growth assays using cells from (F). J) Quantification of I. Error bars were calculated as mean+/‐SD, *n* = 3. *p < 0.05 (one‐way ANOVA test). K) IB analysis of WCL derived from shSTING‐tet‐on A498 cells treated with or without 1 µg ml^−1^ doxycycline. L) Representative images for 2D colony formation from K. M) quantification of L. N,O) Mouse xenograft experiments were performed with indicated A498 cells. 66 days post‐injection, mice were sacrificed, and tumors were dissected (N) and weighed (O). *n* = 8. *p<0.05 (one‐way ANOVA test). P,Q) Mouse xenograft experiments were performed with teton‐shSTING‐2 A498 cells. Doxycycline was administrated to mice at day 15 post‐injection. 39 days post‐injection, mice were sacrificed, and tumors were dissected (P) and weighed (Q). *n* = 9. *p<0.05 (one‐way ANOVA test). R) IB analyses of WCL derived from WT or S366A‐knockin A498 cells treated with 5 µg mL^−1^ ISD90 for indicated periods. S,U) Representative images for 2D colony formation or 3D soft agar growth assays using indicated cells. T,V,W) Quantification of S, U. Error bars were calculated as mean+/‐SD, *n* = 3. *p < 0.05 (one‐way ANOVA test).

A recent study profiling cGAS/STING expression in human tumors confirmed reduced STING expression in most cancer types, but a few tumors including kidney cancer (KIRC) showed STING overexpression.^[^ ^33^ ^]^ Our analyses of the TCGA dataset identified gene amplification of *STING* (*TMEM173, STING1*) but not *cGAS (MB21D1)* (**Figure** **1**A), as well as increased STING mRNA levels (≈10%) in clear cell renal cell carcinoma (ccRCC) (Figure 1B). Furthermore, we observed increased STING protein expression in RCC tumors compared to the normal adjacent tissues (Figure 1C; Figure S1A, Supporting Information), and higher levels of STING protein expression correlated with poor survival (Figure 1D). These clinical data in RCC challenge the common notion that STING facilitates anti‐tumor immunity. We profiled STING protein expression in a panel of RCC cell lines and found that STING protein expression varied among these cell lines with A498 and RCC10 cell lines showing the highest STING expression (Figure 1E). Depletion of endogenous STING in RCC and non‐RCC cell lines by three independent shRNAs (Figure 1F) led to reduced ability of colony formation (Figure 1G,H) and decreased 3D anchorage‐independent growth in vitro (Figure 1I,J) with the only exception of HEK293 cells (Figure 1G,H). In contrast, STING deletion in HeLa cells (Figure S1B,C, Supporting Information) and BPH1 cells (Figure S1D–F, Supporting Information) did not significantly decrease cell growth in vitro, suggesting that STING may have a unique role in sustaining the growth of RCC. Of note, tumor growth inhibition in STING‐depleted tumor cells could be partially rescued by re‐expressing STING (Figure S1G–I, Supporting Information), indicating that the observed growth inhibition mediated by STING shRNAs may not be due to off‐target effects. We also isolated STING‐deleted A498 single clones obtained by CRISPR (Figure S1J, Supporting Information) and found that most of the clones with deleted STING showed significantly reduced growth (Figure S1K, Supporting Information). In addition, doxycycline‐induced depletion of STING (Figure 1K) also reduced A498 colony formation in vitro (Figure 1L,M). We next engrafted control and STING‐depleted A498 cells in nude mice and observed that STING depletion significantly reduced tumor formation (Figure 1N,O; Figure S1L,M, Supporting Information). STING depletion‐induced RCC cell growth retardation was also confirmed in 786‐O cells (Figure S1N–Q, Supporting Information). In addition, to examine effects of STING loss in RCC tumor progression, we xenografted A498 cells in nude mice and induced STING depletion in established A498 tumors in a doxycycline‐dependent manner. STING depletion in established A498 tumors also retarded tumor growth in mice (Figure 1P,Q; Figure S1R,S, Supporting Information). Overall, these data indicate that STING is directly involved in supporting the growth of RCC, and this effect may be independent of its canonical innate immune function given that there is a lack of an immune environment in these data obtained from in vitro cell culture and immune‐deficient nude mice.

### 2.2 Depletion of STING Reduces RCC Cell Growth Independent of Innate Immunity

To further examine a possible innate immunity‐independent function of STING in governing RCC growth, we depleted cGAS, the upstream activator of STING in cytosolic DNA sensing, in either A498 or RCC10 cells by shRNAs (Figure S2A, Supporting Information) or sgRNAs (Figure S2B,C, Supporting Information). In sharp contrast to STING depletion, depletion of cGAS by either approach did not reduce cell growth in vitro (Figure S2D–G, Supporting Information). In addition, depletion of the major STING downstream effector IRF3 by sgRNAs in either A498 or RCC10 cells (Figure S2H,I, Supporting Information) also minimally affected cell growth in vitro (Figure S2J,K, Supporting Information). Furthermore, we generated *STING‐S366A* knockin A498 cells by CRISPR (Figure S2L, Supporting Information) that lack IRF3 phosphorylation in response to STING activation (Figure 1R) critical for the canonical STING‐mediated innate immune response.^[^ ^34^ ^]^ *STING‐S366A* knockin A498 cells did not display observable changes in cell growth in vitro (Figure 1S,T) compared with parental A498 cells, while STING depletion in *STING‐S366A* knockin A498 cells (Figure S2M, Supporting Information) similarly caused inhibition of cell growth in both 2D and 3D growth assays (Figure 1U–W). Overall, these data suggest that STING promotes the growth of RCC independently from the canonical IRF3‐ innate immune pathway.

### 2.3 Depletion of STING Causes RCC Cell Cycle Arrest and Senescence Via Deregulated Mitochondrial Calcium Homeostasis

**Figure 2:**
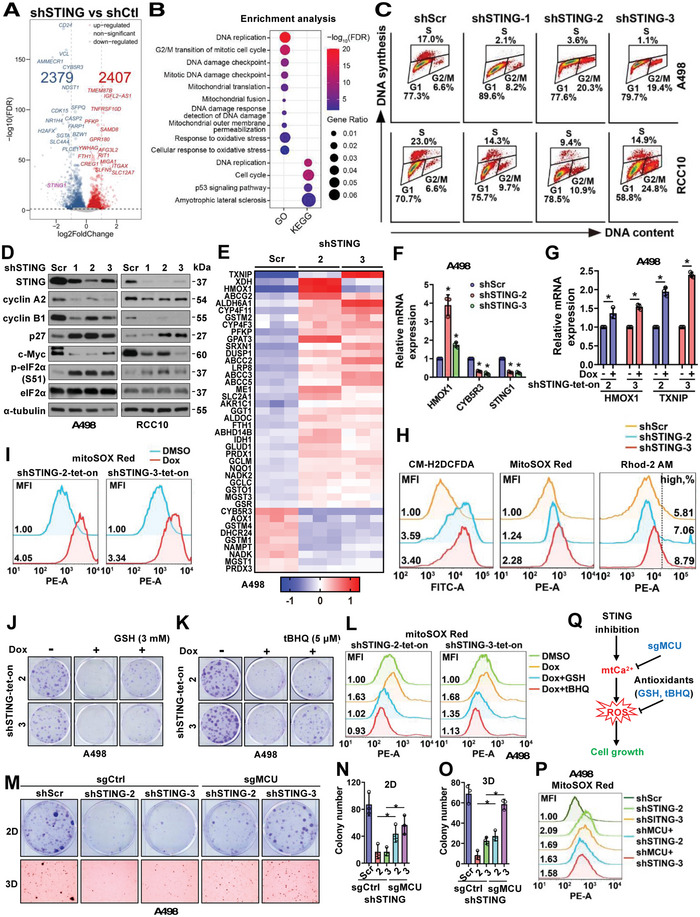
STING depletion in RCC cells increases mitochondria ROS/Ca^2+^ and cell cycle deregulation. A) A volcano plot indicating 2379 down‐regulated genes and 2407 upregulated genes upon STING depletion in A498 cells by RNA‐Seq analyses. B) Pathway analyses by GO and KEGG as indicated on cellular processes. Fold changes are represented by the sizes of dots and statistical significance is indicated by warm/cold color as indicated. C) Cell cycle analyses by FACS using indicated RCC cells. D) IB analyses of WCL derived from indicated RCC cells depleted of STING. E) A heatmap for indicated statistically significant altered genes associated with Nrf2 signaling from RNA‐Seq analyses in indicated A498 cells. F,G), RT‐PCR analyses of mRNA changes in indicated A498 cells. Error bars were calculated as mean+/‐SD, *n* = 3. *p < 0.05 (one‐way ANOVA test). H,I) FACS analyses of cellular ROS (CM‐H2DCFDA), mitochondria ROS (MitoSOX Red), and mitochondria Ca^2+^ (Rhod‐2 AM) in indicated A498 cells. Mean fluorescent intensity (MFI) or high calcium percentage is shown in each histogram panel. J,K,M) Representative images for 2D colony formation assays or 3D soft agar assay using indicated cells. GSH, 3 mM; tBHQ, 5 µM. L,P) FACS analyses of mitochondria ROS (MitoSOX Red) in indicated A498 cells. N,O) Quantification of M. Error bars were calculated as mean+/‐SD, *n* = 3. *p < 0.05 (one‐way ANOVA test). Q) Schematic model shows that STING inhibition increases mitochondria ROS and suppresses cell growth, which can be rescued by antioxidants like GSH and tBHQ.

To determine the intracellular changes associated with STING depletion in A498 cells that contribute to cell growth inhibition, we performed RNA‐Seq analyses in three independent STING‐depleted A498 tumor cells (Figure S3A, Supporting Information). Expression of 2407 and 2379 genes were upregulated and downregulated, respectively, in STING‐depleted cells compared with control cells (**Figure** **2**A). Pathway enrichment analyses identified cell cycle arrest and DNA damage response as top hits (Figure 2B; Figure S3B, Supporting Information). FACS analyses of cell cycle progression confirmed that STING depletion strongly reduced the proportion of cells in S phase with corresponding increases in G1 and G2/M (Figure 2C), accompanied by reduced expression of cell cycle markers such as cyclin A2 and cyclin B1 (Figure 2D and Figure S3C). Accompanying the cell cycle arrest, we observed increased cellular senescence in STING‐depleted A498 or RCC10 tumor cells (Figure S3D,E, Supporting Information). To further understand how STING depletion affects cell cycle progression, we found that a cluster of Nrf2 target genes associated with the oxidative stress response was correspondingly changed in STING‐depleted A498 cells (Figure 2E) such as HMOX1, CYB5R3, and TXNIP1 genes (Figure 2F–G). In ccRCC patient cohorts,^[^ ^35^ ^]^ increased *STING* mRNA levels positively correlated with *CYB5R3* mRNA expression (Figure S3F, Supporting Information), further supporting the potential role of STING in regulating oxidative stress responses in RCC. In addition, expression of genes associated with the response to oxidative stress and mitochondrial function was also altered (Figure 2B). Indeed, in A498 cells, we observed that upon STING depletion, not only cellular ROS (evidenced by CM‐H2DCFDA, Figure 2H) but also mitochondrial ROS (by MitoSOX Red, Figure 2H,I) were increased. Considering the fact that cancer cells generate hydrogen peroxide and reactive oxygen species (ROS)^[^ ^36^ ^]^ that further cause genome instability^[^ ^37^ ^]^ and cell cycle arrest,^[^ ^38^ ^]^ we thought increased ROS may contribute to the observed cell cycle arrest and DNA damage upon STING depletion in A498 cells. To test this hypothesis, we attempted to reduce cellular ROS levels. Treatment with an antioxidant GSH (glutathione, Figure 2J; Figure S3G, Supporting Information) or tBHQ (tert‐butylhydroquinone, Figure 2K) partially rescued STING depletion‐induced growth reduction in A498 cells, which might be due to the ability of either GSH or tBHQ treatment in reducing mitochondrial ROS as evidenced by MitoSOX Red (Figure 2L; Figure S3H, Supporting Information). Thus, we thought STING depletion in A498 cells increases cellular ROS levels, leading to cell cycle arrest and a deregulated DNA damage response. However, how STING deficiency upregulates cellular and mitochondrial ROS remains unknown.

Calcium dysregulation has been shown to impair ER and mitochondria function resulting in ROS increase.^[^ ^39^ ^]^ The gain‐of‐function STING‐N153S mutant was reported to cause T cell death by disrupting calcium homeostasis.^[^ ^40^ ^]^ In A498 cells, we observed that upon STING depletion, not only cellular ROS (evidenced by CM‐H2DCFDA) and mitochondrial ROS (by MitoSOX Red), but also mitochondrial calcium levels (by Rhod‐2 AM) (Figure 2H) were increased. Incubation with the calcium chelator BAPTA‐AM that suppresses cellular calcium signaling did not rescue STING depletion‐induced A498 cell growth reduction (Figure S3I, Supporting Information), suggesting that increased mitochondrial calcium and ROS rather than ER calcium signaling may contribute to inhibiting cell growth upon STING depletion. We thus depleted the mitochondrial inner membrane‐anchored calcium uniporter MCU^[^ ^41^ ^]^ by either sgRNA (Figure S3J, Supporting Information) or shRNA (Figure S3K, Supporting Information) to attenuate mitochondrial calcium uptake. We found that MCU depletion partially rescued STING depletion‐induced cell growth inhibition in vitro (Figure 2M–O; Figure S3L–N, Supporting Information), which correlated with reduced mitochondrial ROS (Figure 2P), suggesting that mitochondrial ROS/calcium increases may partially explain observed cell growth inhibition in STING‐depleted tumor cells (Figure S3O, Supporting Information).

### 2.4 STING Binds Mitochondrial Calcium Transporter VDAC2 to Control Mitochondrial Calcium Homeostasis

**Figure 3:**
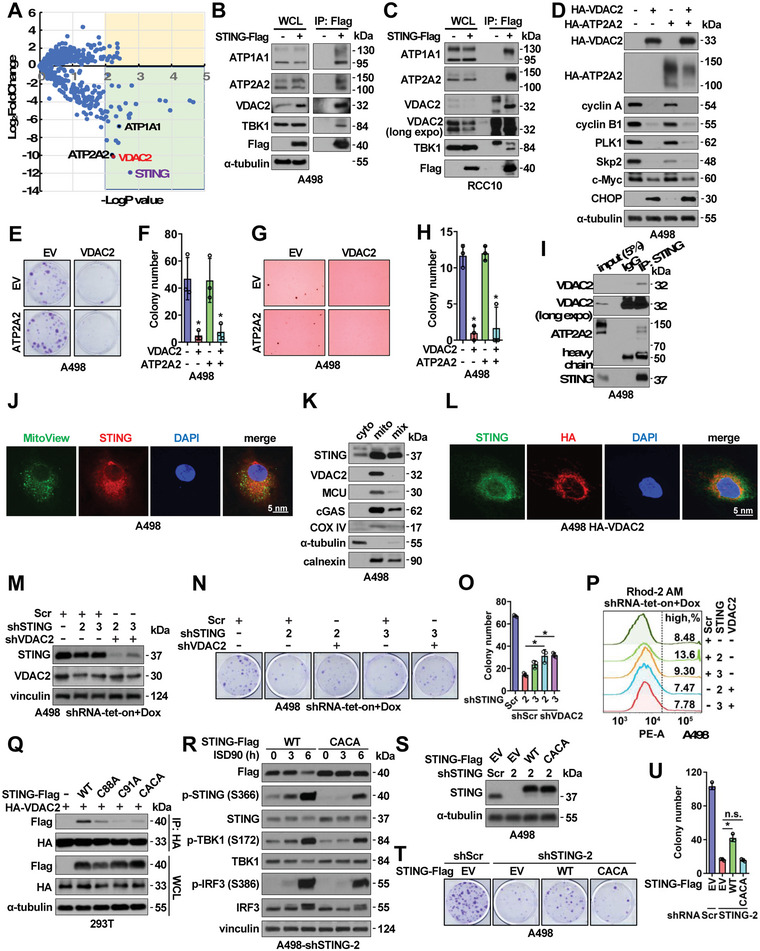
STING binds VDAC2 to maintain RCC cell growth. A) A volcano plot showing major STING binding partners from proteomic studies. STING is labeled in purple, and three calcium transporters are as labeled. B,C) IB analyses of Flag‐IP and WCL derived from either A498 (B) or RCC10 (C) stably expressing Flag‐STING through lentiviral infection. D) IB analyses of WCL from A498 cells stably expressing indicated molecules. E–H) Representative images for 2D colony formation assays E or 3D soft agar assays G using cells from D and quantified in F, H. Error bars were calculated as mean+/‐SD, *n* = 3. *p < 0.05 (one‐way ANOVA test). I) IB analyses of endogenous STING‐IP and inputs from A498 cells. J) Representative immunofluorescent images using A498 cells immune‐stained with an anti‐STING antibody, mitoView, and DAPI. Scale bar represents 5 µm. K) IB analyses of fractionated A498 cell lysates. L) Representative immunofluorescent images using A498 cells stably expressing HA‐VDAC2 by lentiviral infection immune‐stained with an anti‐STING antibody, an anti‐HA antibody, and DAPI. Scale bar represents 5 µm. M) IB analyses of WCL derived from indicated A498 cells depleted of indicated targets by doxycycline treatment. N) Representative images for 2D colony formation assays using cells from M and quantified in O. Error bars were calculated as mean+/‐SD, *n* = 3. *p < 0.05 (one‐way ANOVA test). P) FACS analyses of mitochondria Ca^2+^ (Rhod‐2 AM) in indicated A498 cells. Q) IB analyses of HA‐IP and WCL derived from HEK293T cells transfected with indicated DNA constructs. R) IB analyses of WCL derived from ISD90‐treated A498 cells stably expressing WT or CACA (C88A/C91A)‐STING. S) IB analyses of WCL derived from indicated A498 cells. Where indicated, WT or C88A/C91A (CACA)‐STING containing lentiviral viruses were used to generate A498 stable cells, then endogenous STING was depleted by indicated shRNAs via lentiviral infections. T) Representative images for 2D colony formation assays using cells from S and quantified in U. Error bars were calculated as mean+/‐SD, *n* = 3. *p < 0.05 (one‐way ANOVA test).

To further understand how STING governs ccRCC growth by regulating mitochondrial ROS/calcium signaling, we performed proteomics studies to identify potential new STING binding partners. Upon removing proteins identified only by site, reverses, and potential contaminants, as well as data normalization and statistical comparisons, functional enrichment analyses suggested that membrane channel proteins were the most abundant STING binding partners except those localized in ER (Figure S4A, Supporting Information). Among them, three calcium transporters/pumps were identified, including VDAC2 (Voltage‐dependent anion channel 2 located on mitochondria outer‐membrane),^[^ ^42^ ^]^ ATP2A2 (SERCA Ca‐ATPase located on ER membrane),^[^ ^43^ ^]^ and ATP1A1 (sodium/potassium‐transporting ATPase subunit alpha‐1 located on plasma membrane) (Figure 3A ). Binding of STING with VDAC2, ATP2A2, and ATP1A1 was confirmed in both A498 (**Figure** **3**B; Figure S4B) and RCC10 (Figure 3C; Figure S4C, Supporting Information) tumor cells. Of note, overexpression of VDAC2, but not ATP2A2, in A498 cells reduced cyclin A2 and cyclin B1 protein levels (Figure 3D) and reduced A498 cell growth in both 2D (Figure 3E,F) and 3D (Figure 3G,H) culture assays mimicking the effects caused by STING depletion (Figure 1G). STING interaction with VDAC2 was further confirmed at endogenous levels in A498 cells (Figure 3I). Overall, these data suggest that the mitochondrial outer membrane calcium transporter VDAC2 is a new STING binding partner and the STING‐VDAC2 interaction may regulate mitochondrial ROS/calcium levels.

STING is primarily located in ER and traffics to Golgi/lysosome upon activation.^[^ ^16^ ^]^ Using confocal imaging, we observed that a fraction of STING co‐localized with MitoView as a mitochondria marker in A498 tumor cells (Figure 3J), and STING was detected in purified mitochondrial fractions (Figure 3K). Moreover, colocalization of STING with VDAC2 was observed in A498 cells (Figure 3L). The innate immune signaling‐deficient STING‐S366A mutant (Figure S4D, Supporting Information) retained a comparable interaction with VDAC2 (Figure S4E, Supporting Information), indicating that STING‐VDAC2 interaction is not dependent on the canonical STING pathway. We generated truncated forms of the STING proteins (Figure S4F, Supporting Information) and found the TM (transmembrane) domains (Figure S4G, Supporting Information), especially the TM2‐4 (aa 42–150) are involved in the VDAC2 binding (Figure S4G, Supporting Information). On the other hand, three VDAC2 cancerous mutations were observed in more than 2 cancer patients including VDAC2‐Q101K, S128A, and L136F from cBioPortal (Figure S4H, Supporting Information). Interestingly, all these cancerous VDAC2 mutants displayed an increased binding affinity with STING, while a VDAC2‐E84Q mutant previously identified in deficient binding ceramides^[^ ^44^ ^]^ reduced binding with STING (Figure S4I, Supporting Information). As a consequence, unlike WT‐VDAC2, which suppressed cyclin A2 and cyclin B1 expression (Figure S4J) and subsequently reduced cell growth (Figure S4K, Supporting Information), the E84Q‐VDAC2 mutant failed to do so partially due to its deficiency in interacting with STING. Moreover, we found that depletion of VDAC2 partially rescued STING depletion‐induced A498 cell growth retardation (Figure 3M–O), which is largely due to the rescue of mitochondrial calcium signaling by VDAC2 depletion (Figure 3P).

Considering that STING C88/C91 residues within the TM2‐4 region have been shown to be palmitoylated and cause STING oligomerization^[^ ^45^ ^]^ and translocation to Golgi,^[^ ^46^ ^]^ we tested if these amino acids are critical in mediating the STING‐VDAC2 interaction. Each single CA mutant, and to a higher extent, the CACA‐STING double mutant showed reduced binding with VDAC2 in cells (Figure 3Q; Figure S4L, Supporting Information). The STING‐CACA mutant did not significantly affect the activation of cGAS/STING signaling induced by ISD90 in A498 cells (Figure 3R). We reconstituted STING‐depleted A498 cells with WT‐STING or the STING‐CACA mutant (Figure 3S) and found that only the WT‐STING partially rescued A498 cell growth in vitro (Figure 3T–U), further indicating that the STING‐VDAC2 interaction in the mitochondria is critical in suppressing VDAC2 functions and promoting RCC cell growth. Consistent with this notion, re‐expressing WT‐STING partially rescued the mitochondrial ROS/calcium levels in STING‐depleted tumor cells, while CACA‐STING failed to do so (Figure S4M, Supporting Information). Furthermore, in STING and VDAC2 double‐depleted A498 cells, compared with WT‐VDAC2/WT‐STING, reconstitution with WT‐VDAC2/CACA‐STING (Figure S4N, Supporting Information) showed an increased mitochondrial calcium level due to the inability of CACA‐STING in binding and suppressing VDAC2 (Figure S4O, Supporting Information). On the other hand, E84Q‐VDAC2 was reported to be deficient in transporting calcium into mitochondria^[^ ^47^ ^]^ and WT‐STING/E84Q‐VDAC2 reconstitution showed WT‐STING failed to bind and suppress E84Q‐VDAC2 induced reduction in mitochondrial calcium levels (Figure S4O, Supporting Information). These data further support the importance of STING/VDAC2 interactions in governing mitochondrial calcium homeostasis.

### 2.5 STING Binding to VDAC2 Disrupts MERC (Mitochondria‐Endoplasmic Reticulum Contact) and Reduces Calcium Transfer into Mitochondria

**Figure 4:**
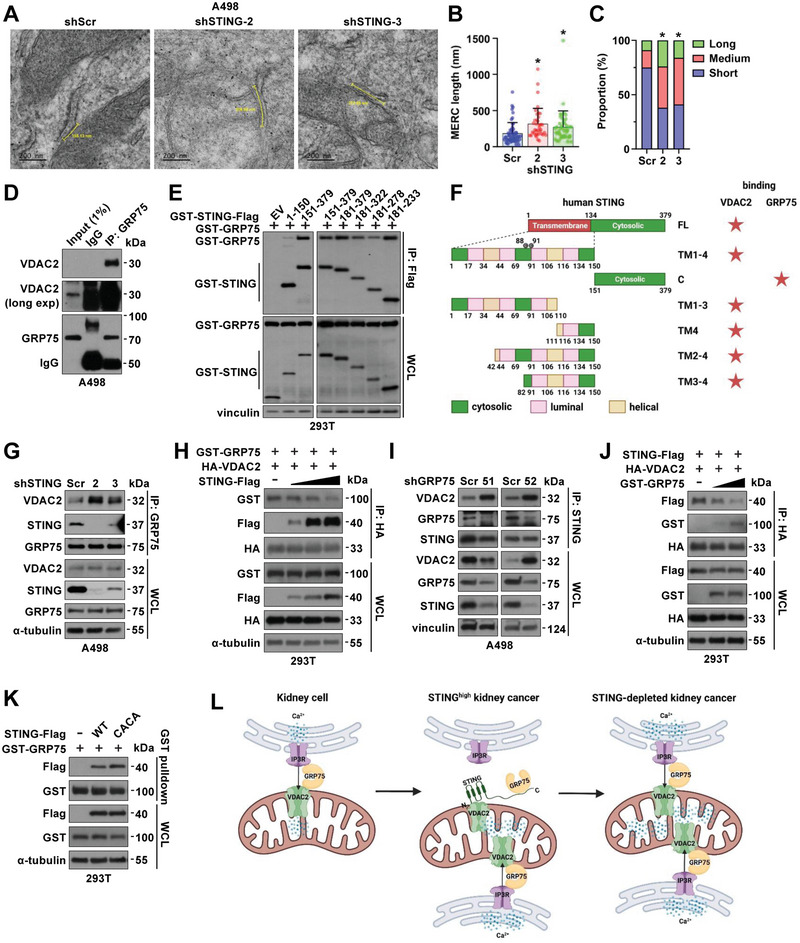
STING disrupts MERC and mitochondrial Ca^2+^ homeostasis through interfering with VDAC2/GRP75 interactions. A) Representative electron microscope images of MERC in shScr and shSTING A498 cells 4 days after virus infection. The scale bar represents 200 nm. Shorter yellow lines between mitochondria outer membrane and ER membrane indicate 30 nm MERC distance. Longer yellow curves or lines between shorter lines indicate MERC length. B,C) Quantification of average MERC length (B) and distribution of MERC length (C) in shScr (*n* = 56), shSTING‐2 (*n* = 37), and shSTING‐3 (*n* = 44) A498 cells. Error bars were calculated as mean+/‐SD. *p < 0.05 (one‐way ANOVA test). Short, medium and long MERC length were defined as length that is <200 nm, 200–400 nm, and >400 nm, respectively. Chi‐square test, shScr versus shSTING‐2, *χ* ^2^ = 27.9; shScr versus shSTING‐3, *χ* ^2^ = 24.28; *df* = 2, *p < 0.0001. D) IB analyses of GRP75‐IP and WCL from shSTING A498 cells. E) IB analyses of Flag‐IP and WCL from HEK293T cells transfected with indicated DNA constructs. F) A cartoon illustration to indicate distinct binding domains of VDAC2 and GRP75 on STING. G) IB analyses of GRP75‐IP and WCL from shSTING A498 cells. H,J) IB analyses of HA‐IP and WCL derived from HEK293T cells transfected with indicated DNA constructs. I) IB analyses of STING‐IP and WCL from shGRP75 A498 cells. K) IB analyses of GST‐pulldown and WCL from HEK293T cells transfected with indicated DNA constructs. L) A schematic model showing abundant STING in RCC binds VDAC2 and prevents formation of ER‐mitochondria tethering complex VDAC2‐GRP75, thus maintaining low levels of mitochondria calcium uptake. STING deficiency leads to enhanced VDAC2‐GRP75 interactions to increase MERC facilitating mitochondrial Ca^2+^ transfer to increase mitochondrial ROS and cellular ROS and leading to RCC cell growth suppression.

We next went on to examine how STING binding to VDAC2 regulates VDAC2 function in RCC. We found that STING depletion in A498 cells did not significantly affect activities of Ca^2+^‐related ATPases (Figure S5A, Supporting Information), and did not significantly affect VDAC2 dimer formation (Figure S5B, Supporting Information), which is critical for VDAC2 function as a mitochondrial calcium transporter.^[^ ^48^ ^]^ Instead, we observed that STING depletion significantly enhanced MERC (mitochondria‐ER contact, **Figure** **4**A–C), which has been shown to be critical in regulating lipid generation, Ca^2+^ transfer, trafficking, and mitochondrial function such as biogenesis.^[^ ^49^ ^]^ Recently, MERC has also been shown to mediate ER‐mitochondria tethering, metabolite exchanges, redox status and serve as a multiple signaling platform to regulate cell proliferation, senescence, or aging.^[^ ^50^ ^]^ MERC sites are important to locally mediate calcium signals between ER protein IP3R (IP3 receptors) and mitochondrial calcium uniporters (such as VDACs). In this process, GRP75 (glucose‐regulated protein 75) at the mitochondria‐associated membrane (MAM) bridges IR3R interaction with VDACs. Given we observed STING depletion induced increased Ca^2+^ flux into mitochondria and upregulated mito‐ROS levels (Figure 2H), we examined if STING depletion enhanced MERC is due to STING binding and blocking VDAC2 interactions with GRP75. Consistent with previous reports indicating that ER‐mitochondria contacts regulate mitochondrial homeostasis and calcium transfer from ER to mitochondria,^[^ ^51^, ^52^ ^]^ we observed that VDAC2 bound GRP75 at endogenous levels in A498 cells (Figure 4D). Notably, treatment of A498 cells with ISD90 that activates innate immune responses did not affect GRP75 binding to VDAC2 (Figure S5C, Supporting Information), suggesting that STING may regulate ER‐mitochondria contacts independent of its canonical function in innate immunity. Unlike VDAC2 binding to STING‐TM(2‐4) (Figure S5H, Supporting Information), GRP75 might largely interact with the STING‐cytosolic domain (Figure 4E–F), although it may be due to that TM‐deleted STING residing in cytoplasm with a higher chance to interact with cytosolic VDAC2. Nonetheless, depletion of STING led to enhanced GRP75 binding with VDAC2 (Figure 4G), and ectopic STING expression in a dose‐dependent manner disrupted VDAC2 binding to GRP75 (Figure 4H). Depletion of GRP75 also promoted STING binding to VDAC2 (Figure 4I) and ectopic GRP75 expression attenuated this interaction (Figure 4J). On the other hand, ectopic VDAC2 expression did not significantly affect STING binding to GRP75 (Figure S5D, Supporting Information). Moreover, neither STING interaction with GRP75 nor GRP75 binding to VDAC2 was affected by loss of STING‐S366 phosphorylation (Figure S5E,F), or depletion of cGAS (Figure S5G,H, Supporting Information), suggesting an innate immune function of STING is unlikely to regulate VDAC2/GRP75 interactions in cells. In A498 cells, cell fractionation assays identified fractions with expression of STING, VDAC2, and GRP75 (Figure S5I, Supporting Information), suggesting a possible regulatory mechanism among these proteins. Furthermore, the VDAC2 binding‐deficient STING‐CACA mutant displayed a comparable binding as STING‐WT to GRP75 (Figure 4K). These data suggest that STING may compete with GRP75 to bind VDAC2, thus STING depletion enhances GRP75/VDAC2 contacts leading to increased Ca^2+^ transfer from ER to mitochondria (Figure 4L).

### 2.6 STING Depletion Leads to Mitochondrial Dysfunction and Suppresses mTORC1/S6K Signaling to Inhibit RCC Cell Growth

**Figure 5:**
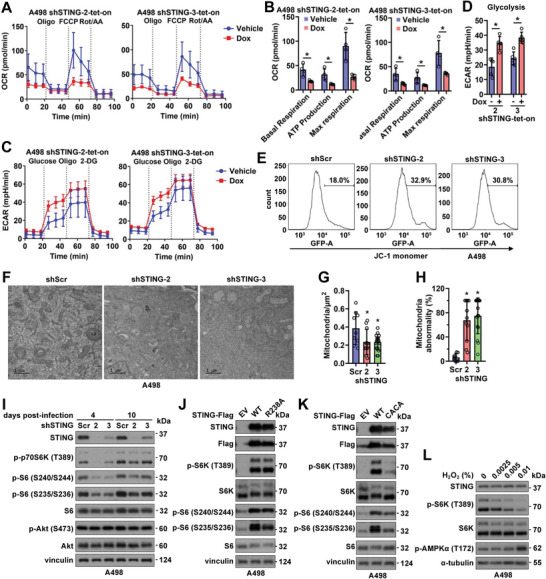
STING depletion leads to mitochondrial abnormality, reduced ATP production, and suppression of mTORC1/S6K signaling to inhibit RCC growth. A,C) Normalized OCR (A) and ECAR plots (C) using shSTING‐tet‐on A498 cells with or without 1 mg mL^−1^ doxycycline treatment for 4 days. Quantifications are shown in B and D. Error bars were calculated as mean+/‐SD, *n* = 5 or 4. *p < 0.05 (one‐way ANOVA test). E) FACS analyses of mitochondria potential (JC‐1 monomer) in indicated A498 cells. F) Representative images for EM (electron microscopy) analyses of A498 cells 4 days post‐infection by indicated viruses. The scale bar represents 1 µM. G,H) Quantification of the number of mitochondria in each µm^2^ in G and percentage of abnormal mitochondria in H. Error bars were calculated as mean+/‐SD, *n* = 9 for shScr, *n* = 11 for shSTING‐2, *n* = 16 for shSTING‐3. *p < 0.05 (one‐way ANOVA test). I) IB analysis of WCL derived from A498 cells infected with indicated viruses for either 4 days or 10 days. J,K) IB analysis of WCL from A498 cells stably expressing indicated Flag‐STING by lentiviral infection. L) IB analysis of WCL derived from A498 cells treated with indicated concentrations of H_2_O_2_ for 6 h before cell collection.

Considering MERC is critical for mitochondria function, we went on to examine bioenergetics in mitochondria, especially OCR (oxygen consumption rate) and ECAR (extracellular acidification rate) by seahorse. STING‐depleted A498 cells displayed a decreased basal respiration rate compared with control cells and a decreased ATP‐driven synthesis ability (**Figure** **5**A,B; Figure S6A,B, Supporting Information), which could be largely rescued by re‐expressing WT‐STING (Figure S6C–E, Supporting Information). These data suggest that increased mitochondrial calcium transport upon STING depletion (Figure 2H) may cause reduced basal respiration and ATP synthesis (Figure 5B), therefore slowing down cell proliferation. On the other hand, STING depletion increased rates of glycolysis to compensate for energy shortage (Figure 5C,D; Figure S6F–G, Supporting Information). As a result of imbalance of mitochondria Ca^2+^ homeostasis, STING depletion significantly decreased the mitochondrial membrane potential (Δ*Ψ*m, by JC‐1 monomer, Figure 5E). Considering normal mitochondria potential plays a critical role in controlling mitochondrial homeostasis through eliminating unhealthy mitochondria, we observed that depletion of STING in A498 cells resulted in both reduced numbers of mitochondrion and increased mitochondria abnormality (Figure 5F–H) in an EM (electron microscopy) experiment.

Next, we went on to examine how increased mitochondrial calcium/ROS negatively regulates RCC growth. We found that mTORC1 but not mTORC2 signaling was suppressed upon STING depletion in A498 cells (Figure 5I). Moreover, reduced S6K activity (evidenced by S6‐pS240/S244 and S6‐pS235/236) but not 4E‐BP1 phosphorylation downstream of mTORC1 was observed (Figure 5I). These data suggest that STING may play a role in maintaining mTORC1/S6K signaling in RCC to regulate cell growth. We observed STING bound various mTOR complex components including G*β*L, Raptor, and Sin1 (Figure S6H, Supporting Information), in which Raptor mTORC1 specific. While STING neither regulated Raptor binding with mTOR that is essential for mTORC1 complex formation and function (Figure S6I, Supporting Information), nor affected Raptor binding with S6K1 (Figure S6J,K, Supporting Information). These data indicate that STING might not directly regulate mTORC1/S6K activity, but rather indirectly through regulating mitochondrial calcium/ROS.^[^ ^53^ ^]^ Consistent with this notion, we found that both WT‐ and an R238A‐STING (deficient in binding 2′3′‐cGAMP thus deficient in STING innate immune function) could promote mTORC1/S6K activation in A498 cells (Figure 5J), while CACA‐STING (deficient in binding VDAC2) failed to stimulate mTORC1/S6K activation (Figure 5K). Ectopic STING expression also facilitated mTORC1 activation in HEK293T or 786‐O cells (Figure S6L, Supporting Information). Moreover, treating A498 cells with H_2_O_2_ to mimic STING depletion by increasing cellular ROS also led to an H_2_O_2_ dose‐dependent decrease of mTORC1/S6K activation (Figure 5L), where STING or VDAC2 oligomerization was not significantly affected (Figure S6M, Supporting Information). In addition, VDAC2 expression suppressed WT‐ or R238A‐STING‐induced mTORC1 activation (Figure S6N, Supporting Information) and subsequently compromised STING‐mediated cell growth (Figure S6O,P, Supporting Information). Together, these data suggest that STING depletion increases mitochondrial calcium/ROS, leading to mitochondrial dysfunction and suppression of mTORC1/S6K signaling, therefore negatively regulating RCC cell growth. Examinations of clinical RCC patient tumors compared with normal adjacent tissues confirmed a positive correlation of increased STING expression in tumors with increased mTORC1/S6K activation (Figure S7A, Supporting Information). In addition, increased *STING* mRNA levels negatively correlated with mRNA expression of oxidative stress‐responsive genes including *ABCG2* ^[^ ^54^ ^]^ and *ALDH6A1* ^[^ ^55^ ^]^ (Figure S7B,C, Supporting Information) in ccRCC patient cohorts^[^ ^35^ ^]^ that might be controlled by mTORC1. Notably, if STING similarly regulates RCC metabolism in vivo warrants further investigations given differences in metabolic changes from in vitro and in vivo studies have been observed.^[^ ^56^ ^]^

### 2.7 STING Palmitoylation Facilitates VDAC2 Binding and Maintains RCC Growth

**Figure 6:**
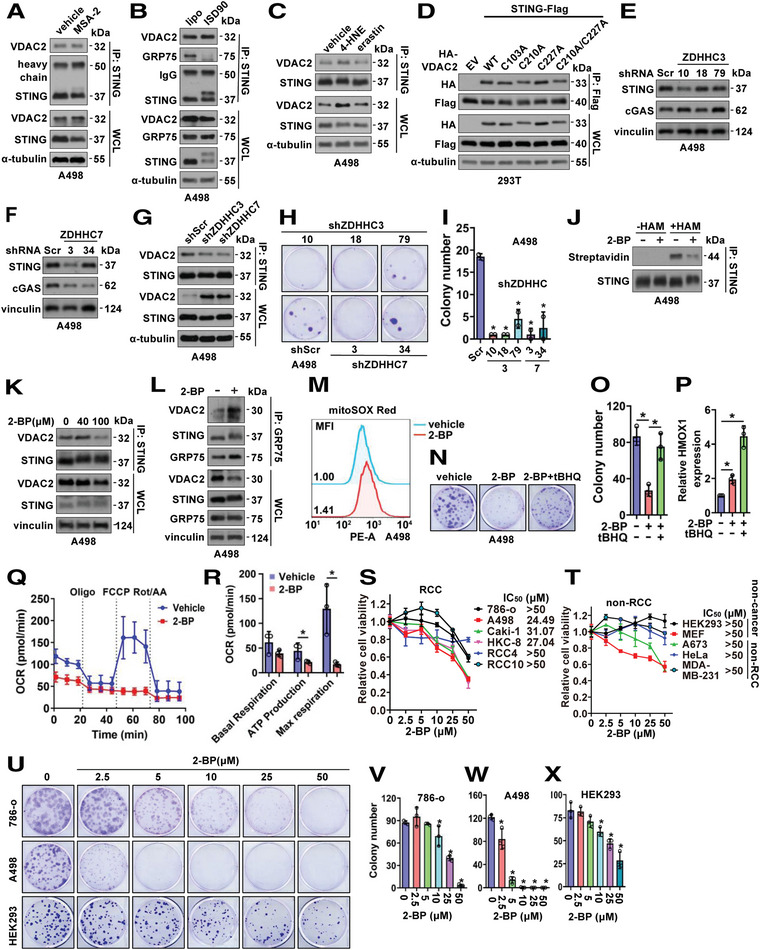
Inhibition of STING palmitoylation suppresses RCC cell growth. A–C) IB analyses of STING‐IP and WCL derived from A498 cells with indicated treatments. Where indicated, MSA‐2 (50 µM, 24 h in A), ISD90 (5 µg mL^−1^, 4 h in B), 4‐HNE (50 µM, 24 h in C), erastin (20 µM, 24 h in C) were used to treat A498 cells. D) IB analyses of Flag‐IP and WCL from HEK293T cells transfected with indicated DNA constructs. E,F) IB analyses of A498 cells depleted of indicated ZDHHC by lentiviral infection and selected with 2 µg mL^−1^ puromycin for 72 h to eliminate non‐infected cells. G) IB analyses of STING‐IP and WCL derived from indicated A498 cells. H) Representative images for 2D colony formation assays using cells from (E,F) and quantified in I. Error bars were calculated as mean+/‐SD, *n* = 2. *p < 0.05 (one‐way ANOVA test). J) IB analyses of STING immune‐precipitates from control or 2‐BP treated A498 cells. K) IB analyses of STING‐IP and WCL derived from A498 cells treated with indicated doses of 2‐BP for 12 h before cell collection. L) IB analyses of GRP75‐IP and WCL derived from A498 cells treated with 100 µM of 2‐BP for 12 h before cell collection. M) FACS analyses of mitochondria ROS levels (MitoSOX Red) in indicated A498 cells. N) Representative images for 2D colony formation assays using A498 cells treated with 2.5 µM 2‐BP or 10 µM tBHQ and quantified in O. P) RT‐PCR analysis of HMOX1 mRNA expression in A498 cells treated with 40 µM 2‐BP or 10 µM tBHQ for 48 h. Error bars were calculated as mean+/‐SD, *n* = 3. *p < 0.05 (one‐way ANOVA test). Q) Normalized OCR plots using A498 cells treated with 100 µM 2‐BP for 24 h. Quantification is shown in R. Error bars were calculated as mean+/‐SD, *n* = 3. *p < 0.05 (one‐way ANOVA test). S,T) Cell viability assays using indicated RCC and non‐RCC cells treated with indicated doses of 2‐BP for 72 h before MTT assays. Error bars were calculated as mean+/‐SD, *n* = 3. U) Representative images for 2D colony formation assays using A498, 786‐o, and HEK293 cells treated with indicated doses of 2‐BP (one‐time treatment at 24h post cell seeding) and quantified in V–X) Error bars were calculated as mean+/‐SD, *n* = 3. *p < 0.05 (one‐way ANOVA test).

The critical role of the STING‐VDAC2 interaction in promoting tumor growth in RCC stimulated the development of a therapeutic strategy aiming at disrupting this interaction. We found that the STING agonist MSA‐2^[^ ^57^ ^]^ (**Figure** **6**A; Figure S8A,B, Supporting Information) and ISD90 (Figure 6B; Figure S8C, Supporting Information) that activate innate immunity did not significantly affect STING binding to VDAC2. Inhibiting VDAC2 activity by erastin^[^ ^58^ ^]^ (Figure 6C; Figure S8D,E, Supporting Information) or activating VDAC2 by efsevin^[^ ^59^ ^]^ (Figure S8F, Supporting Information) had minimal effects on modulating STING interaction with VDAC2. Activation of the mitochondrial calcium uniporter by acacetin^[^ ^60^ ^]^ (Figure S8G, Supporting Information), reduction of cellular/mitochondrial ROS levels by GSH (glutathione) (Figure S8H, Supporting Information), and increase of cellular ROS by RSL3^[^ ^61^ ^]^ (Figure S8I, Supporting Information) did not show noticeable effects in modulating STING binding to VDAC2. On the other hand, treatment with BAPTA‐AM (Figure S8J, Supporting Information), a selective intracellular calcium chelator, or H_2_O_2_ leading to increased cellular ROS (Figure S8K, Supporting Information), resulted in increased STING binding to VDAC2. Since STING‐C91A/C88A mutant is deficient in binding VDAC2 (Figure 3Q), and STING palmitoylation on both C91 and C88 were observed to maintain STING function on Golgi,^[^ ^46^ ^]^ we reasoned that inhibiting STING palmitoyltransferase(s) would be an approach to maximize tumor inhibition. In addition, considering that VDAC2 cysteine residue palmitoylation^[^ ^62^ ^]^ and oxidation^[^ ^63^ ^]^ were also previously detected by mass spectrometry, we analyzed if any of these cysteine mutations may affect VDAC2 binding to STING. We generated VDAC2‐C103A, C210A, C227A, and C103A/C210A/C227A (3A) mutants and found none of these mutants significantly affect VDAC2 binding to STING (Figure 6D; Figure S8L,M, Supporting Information). These data suggest that palmitoylation of STING but not VDAC2 may regulate STING binding to VDAC2.

To search for targetable palmitoyltransferase(s), we depleted the protein acyl transferases ZDHHC3 or ZDHHC7 using shRNAs (Figure 6E,F; Figure S8N, Supporting Information). Depletion of ZDHHC3 or ZDHHC7 led to reduced STING binding to VDAC2 in A498 cells (Figure 6G), mimicking effects from STING‐C88A/C91A mutant (Figure 3Q). Moreover, depletion of either ZDHHC3 or ZDHHC7 significantly reduced A498 cell growth in vitro (Figure 6H,I), presumably through increasing mitochondria ROS (Figure S8O, Supporting Information) and mitochondria calcium (Figure S8P, Supporting Information) caused by a deficiency in STING binding to VDAC2. Moreover, WT‐ but not a catalytic dead C160S‐ZDHHC7 promoted STING binding with VDAC2 in cells (Figure S8Q, Supporting Information), suggesting the ZDHHC palmitoylase activity is indispensable for STING binding with VDAC2. ZDHHC inhibitors have been developed with 2‐BP (2‐bromohexadecanoic acid) being the most widely used one showing pan‐inhibitory ability toward ZDHHC family members by forming a covalent bond with cysteines in enzymatic motif.^[^ ^64^, ^65^ ^]^ 2‐BP treatment reduced STING‐palmitoylation at endogenous levels in A498 cells (Figure 6J) and STING‐C88A/C91A displayed reduced levels of palmitoylation compared with STING‐WT in A498 cells (Figure S8R, Supporting Information). Like ZDHHC depletion (Figure 6G), 2‐BP treatment reduced STING binding with VDAC2 (Figure 6K; Figure S8S, Supporting Information) and increased GRP75 binding with VDAC2 (Figure 6L) to facilitate mitochondria ROS accumulation (Figure 6M). Notably, 2‐BP‐mediated ZDHHC inhibition had minimal effects on localizing STING and VDAC2 to mitochondria (Figure S8T, Supporting Information). Moreover, either replacing STING‐WT with STING‐C88A/C91A (Figure S8U, Supporting Information) or depletion of ZDHHC3 or ZDHHC7 (Figure S8V, Supporting Information), disabled effects of 2‐BP in suppressing A498 cell growth. Excitingly, 2‐BP treatment‐reduced A498 cell growth could be rescued by tBHQ (Figure 6N,O) through regulating redox gene expression (Figure 6P) as STING did (Figure 2G). Like STING depletion (Figure 5A), 2‐BP treatment also led to reduced ATP production and respiration in A498 cells (Figure 6Q,R). As a result, 2‐BP treatment caused growth inhibition of RCC cells in vitro, including 786‐o, UMRC6, Caki‐1, HKC‐8, RCC10, and A498 (Figure 6S), while displaying minimal effects in affecting growth of non‐ccRCC cells including HEK293, MEFs (mouse embryonic fibroblast), HeLa and others (Figure 6T). Moreover, 2‐BP also impeded colony formation ability of A498 and 786‐o cells in vitro (Figure 6U–W). Interestingly, at the same dose, compared with A498 and 786‐o cells, HEK293 cells were less sensitive to 2‐BP treatment (Figure 6U,X). Like STING depletion (Figure 5I), 2‐BP treatment led to reduced mTORC1/S6K signaling in A498 cells (Figure S8W, Supporting Information), further supporting mTORC1/S6K as one of the signaling downstream of STING in regulating RCC growth. Overall, these data suggest that 2‐BP by inhibiting the ZDHHC/STING/VDAC2 signaling reduces tumor growth in RCC and may be a promising agent in treating patients with RCC.

### 2.8 2‐BP Displays A Synergy with Sorafenib in Reducing RCC Cell Growth In Vitro and In Vivo

**Figure 7:**
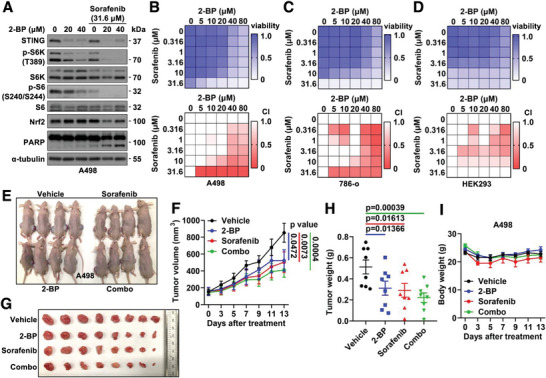
2‐BP and sorafenib exert a synergy in reducing RCC tumor growth. A) IB analyses of WCL from A498 treated with indicated compounds for 48 h before cell collection. B,C,D) Representative heatmaps for cell viability (upper panels) and combination index (CI, lower panels) in A498 cells (B) treated with indicated doses of compounds for 2 days, in 786‐o cells (C) and HEK293 (D) cells treated with indicated doses of compounds for 4 days. White color units in CI heatmaps indicate no synergistic effects. E) Images for nude mice of indicated treatment groups. F) Tumor volume measurements at indicated days after the start of treatment with indicated compounds. Error bars were calculated as mean+/‐SEM, *n* = 8. *p* values are as indicated (two‐way ANOVA followed by Tukey multiple comparison test). G) Isolated tumors from (E) and weighed in H). Error bars were calculated as mean+/‐SEM, *n* = 8. *p* values are as indicated (two‐tailed Student *t*‐test). I) Measurements of mouse body weights at indicated days after treatment of indicated compounds. Error bars were calculated as mean+/‐SEM, *n* = 4.

Considering acquired resistance usually develops with single‐agent treatment,^[^ ^66^ ^]^ and currently various targeted therapies have been approved in treating RCC patients in clinic including tyrosine kinase inhibitors (sunitinib, sorafenib, pazopanib, axitinib, and others), HIF inhibitors (belzutifan) and mTOR inhibitors (temsirolimus and everolimus), we next examined if 2‐BP exerts any synergistic effects with clinically approved agents. To this end, although 2‐BP reduced mTORC1/S6K activation in RCC cells (Figure S8T, Supporting Information), treatment with 2‐BP failed to sensitize A498 or 786‐o cells to rapamycin (Figure S9A,B, Supporting Information), temsirolimus (Figure S9C, Supporting Information), everolimus (Figure S9D, Supporting Information) nor S6K1 inhibitor (S6K‐I) (Figure S9E,F, Supporting Information). Moreover, inhibiting STING innate immunity activity by H‐151 failed to show a synergy with 2‐BP in reducing A498 cell proliferation in vitro (Figure S9G, Supporting Information). On the other hand, addition of sorafenib further inhibited 2‐BP treatment‐mediated mTORC1/S6K activation (**Figure** **7**A) presumably through inhibiting NRF2 signaling (Figure 7A) to reduce levels of cellular anti‐oxidants. As such, 2‐BP displayed a synergy with sorafenib in reducing growth of A498 (Figure 7B) and 786‐o (Figure 7C) cells in vitro, with less effects in suppressing HEK293 cell proliferation (Figure 7D). We then xenografted A498 cells into nude mice and examined effects of 2‐BP or sorafenib alone, or in combination in affecting tumor growth (Figure 7E). Either 2‐BP or sorafenib alone significantly reduced A498 tumor growth, while the combination showed a synergy in further retarding tumor growth (Figure 7F–H). Notably, neither of the treatments influenced mouse body weights (Figure 7I). Together, these data suggest that combination therapy of 2‐BP with sorafenib may improve clinical outcome that warrants further in‐depth investigations.

## 3 Discussion

STING plays an indispensable role in sensing cytosolic DNA to activate innate immunity in host defense against pathogen invasions. In addition to IRF3‐pS396, STING activation governs multiple site phosphorylation of IRF3^[^ ^67^ ^]^ to induce IRF3 mobility changes (Figure 1R) in promoting interferon production. STING also exerts innate immunity‐independent function in T cells to modulate ER stress and calcium homeostasis,^[^ ^29^ ^]^ and in cancer cells to regulate cell cycle and genome instability.^[^ ^30^ ^]^ However, if this STING function relies on STING localization to ER or a similar set of STING downstream binding partners remains unknown. In addition, most previous studies indicate STING depletion favors cell proliferation‐ either through reducing T cell infiltrates to promote immune evasion,^[^ ^20^ ^]^ or via regulating tumor cell cycle depending on the NF‐kB/p53 signaling,^[^ ^30^ ^]^ thus supporting STING as a potential tumor suppressor in cancer.

In this study, we found that STING exerts a novel function in maintaining RCC growth independent of its function in canonical innate immunity control and suggested STING as a new vulnerability in targetedly treating RCC. Distinct from all other reported cancer types, STING depletion in RCC significantly reduced RCC cell growth in vitro and in xenografts largely due to disrupted mitochondrial ROS/calcium homeostasis, leading to cell cycle arrests, cellular senescence, and genome instability. More importantly, we defined VDAC2 as a novel STING binding partner and downstream effector on mitochondria outer membrane. STING binding to VDAC2 disrupted MERC to restrain calcium transfer from ER to mitochondria to maintain calcium homeostasis. Loss of STING or disrupted STING binding to VDAC2 restored VDAC2/GRP75 contacts and MERC function, resulting in increased mitochondria calcium levels and mitochondrial ROS, causing a reduced number of mitochondria and increased mitochondria abnormality. This resulted in decreased mitochondrial membrane potential and increased mitochondria leakage, with reduced ATP production but increased cellular ROS, which subsequently suppressed mTORC1/S6K signaling in causing observed cell cycle arrest and growth retardation. Thus, our mechanistic studies identify STING binding to VDAC2 as a potential therapeutic target in RCC.

To find approaches to disrupt STING binding with VDAC2 in treating RCC, we found palmitoylation of STING‐C88/C91 residues played a critical role in mediating STING binding to VDAC2 and genetic depletion or pharmacological inhibition of ZDHHC palmitoyltransferases efficiently disrupted the STING‐VDAC2 interaction leading to inhibition of cell growth in RCC. Protein palmitoylation inhibitors such as 2‐BP displayed an effect in suppressing RCC cell proliferation. More excitingly, 2‐BP exerted a synergy with sorafenib that is currently used in clinic for RCC treatment in inhibiting RCC cell growth. This provides a rationale to further evaluate the therapeutic potential for the 2‐BP+sorafenib in treating RCC in clinical trials. Moreover, given the vulnerability of targeting STING/VDAC2 binding is not depending on VHL, this combination may benefit RCC patients regardless of VHL status. Since 2‐BP is not specifically inhibiting ZDHHC palmitoyl transferase, developing ZDHHC‐specific inhibitors may improve the therapeutic value. Recently, a STING‐PROTAC synthesized using a STING antagonist as the STING binding warhead has been successfully developed,^[^ ^68^ ^]^ and it will be interesting to test if treating RCC with this STING‐PROTAC reduces RCC proliferation through increasing mitochondrial ROS/calcium homeostasis. Moreover, this reagent provides an opportunity to study effects of STING inactivation in immune‐competent RCC genetic murine models to further examine if STING inhibition could be explored as a treatment direction for RCC patients by balancing STING function in regulating both intrinsic cellular programs such as the one identified here and immune environment. Considering the key function of STING in creating an immune‐friendly microenvironment to facilitate tumor rejection, administration of STING antagonists/PROTACs in cancer treatment would need to be taken with care.

It remains unclear why unlike other cancers including colon cancer,^[^ ^21^ ^]^ gastric cancer,^[^ ^22^ ^]^ and melanoma^[^ ^23^ ^]^ with reduced STING expression, RCC relies on high levels of STING for survival and proliferation. From recent profiling of STING expression in pan‐cancer tissues,^[^ ^33^ ^]^ in addition to RCC, significantly higher levels of STING expression are also observed in STAD (stomach adenocarcinoma) and THCA (thyroid carcinoma). If STING is also a vulnerability in these two types of cancers or if STING regulates mitochondria ROS/calcium homeostasis in STAD and THCA remains an interesting topic for further investigations.

Notably, the heterogeneity of *TMEM173* (STING) gene is observed in different ethnic populations‐ for example, HAQ (R71H‐G230A‐R293Q) is dominant in East Asian, while Africans have no HAQ.^[^ ^69^ ^]^ If these various STING variants affect STING binding with VDAC2 and determine STING dependency for cell growth in cancers beyond RCC warrants further in‐depth investigations.

## 4 Experimental Section

### Materials

Bafilomycin A1 (S1413), cycloheximide (S6611), erastin (S7242), MG132 (S2619), MLN4924 (S7109), S6K1‐I (S2163), tBHQ (S4990) and rapamycin (AY‐22989) were purchased from Selleck. 2‐bromohexadecanoic acid (2‐BP) (238422), puromycin (P8833), hygromycin (H3274), and blasticidin (15205) were purchased from Sigma. l‐Glutathione (reduced) was purchased from Gold Biotechnology (G‐155‐25). 4‐hydroxy Nonenal (4‐HNE) (32100) was purchased from Cayman. Sulfobutylether‐*β*‐Cyclodextrin (HY‐17031), Sorafenib (HY‐10201A), and RSL3 (HY‐100218A) were purchased from MedChemExpress. Everolimus (A8169) was purchased from ApexBio Technology. Temsirolimus (T3574) was purchased from TCI. H‐151 (6675) was purchased from Tocris.

### Antibodies

All antibodies were used at a 1:1000 dilution in TBST buffer with 5% bovine serum albumin or non‐fat milk for western blotting. Anti‐Akt antibody (4691), anti‐ATP2A2 antibody (9580), anti‐calnexin antibody (2679), anti‐cGAS antibody (83623), anti‐CHOP antibody (2895), anti‐c‐Myc antibody (18583), anti‐cyclin B1 antibody (4135), anti‐eIF2*α* antibody (5324), anti‐GRP75 antibody (3593), anti‐HA antibody (3724), anti‐HIF‐1*α* antibody (36169), anti‐IRF3 antibody (4302), anti‐MCU antibody (14997), anti‐p‐4E‐BP1 (Thr37/46) (2855), anti‐p62 antibody (88588), anti‐p70 S6 Kinase (2708), anti‐p‐Akt (Ser473) (4060), anti‐p‐eIF2*α* (Ser51) antibody (3398), anti‐p‐Histone H2A.X (Ser139) antibody (9718), anti‐p‐IRF3 (Ser396) antibody (29047), anti‐PLK1 antibody (4513), anti‐p‐p70 S6 Kinase (Thr389) (9234), anti‐p‐S6 Ribosomal Protein (Ser235/236) (4858), anti‐p‐S6 Ribosomal Protein (Ser240/244) (5364), anti‐p‐STING (Ser366) (50907), anti‐p‐TBK1/NAK (Ser172) (5483), anti‐TBK1 antibody (51872), anti‐Skp2 antibody (2562), anti‐STING antibody (for WB and IP) (13647), anti‐STING antibody (for IF) (90947), anti‐ubiquitin antibody (3936), anti‐VDAC2 antibody (9412), anti‐rabbit IgG, HRP‐linked antibody (7074) and anti‐mouse IgG, HRP‐linked antibody (7076) were obtained from Cell Signaling Technology. Anti‐cyclin A antibody (sc‐751), anti‐GAPDH antibody (sc‐47724), anti‐GST antibody (sc‐459), anti‐NQO1 antibody (sc‐32793), anti‐p27‐antibody (sc‐1641), anti‐PARP‐1‐antibody (sc‐8007), anti‐Ribosomal Protein S6 antibody (sc‐74459) and anti‐vinculin antibody (sc‐25336) were obtained from Santa Cruz Biotechnology. Polyclonal anti‐Flag antibody (F‐7425), monoclonal anti‐Flag antibody (F‐3165, clone M2), anti‐*α*‐tubulin antibody (T‐5168), anti‐Flag agarose beads (A‐2220), anti‐HA agarose beads (A‐2095) and glutathione agarose beads (G4510) were obtained from Millipore Sigma. Anti‐ATP1A1 antibody (14418‐1‐AP), anti‐COX IV antibody (11242‐1‐AP), anti‐HIF‐2*α* antibody (26422‐1‐AP), anti‐Histone H2A.X antibody (10856‐1‐AP) and anti‐VDAC2 antibody (11663‐1‐AP) were obtained from Proteintech. Anti‐Nrf2 antibody (ab62352) was obtained from Abcam. Invitrogen Alexa Fluor 488 goat anti‐rabbit IgG (H+L) (A11034), Invitrogen Alexa Fluor 594 goat anti‐mouse IgG (H+L) (A11032), and Invitrogen Alexa Fluor 594 Donkey anti‐Rabbit IgG (H+L) (A21207) were obtained from ThermoFisher Scientific.

### Human Renal Cell Carcinoma Tumor Specimens

The renal cell carcinoma tumor specimens were obtained from the University of North Carolina at Chapel Hill Lineberger Comprehensive Cancer Center Tissue Procurement Facility reviewed and approved by the Office of Human Research Ethics as described previously.^[^ ^70^, ^71^ ^]^

### Cell Culture and Transfection

Human renal cell carcinoma cell lines 786‐o, A498, ACHN, Caki‐1, RCC4, RCC10, RXF‐393, UMRC2, and UMRC6, human immortalized kidney cell lines HEK293, HEK293T, and HKC‐8, human cervical adenocarcinoma cell line HeLa and human glioblastoma cell line U87MG were cultured in DMEM medium supplemented with 10% FBS. Human benign prostatic hyperplasia cell line BPH‐1 cells were cultured in RPMI‐1640 medium supplemented with 10% FBS. All cell culture media were supplemented with 100 units of penicillin and 100 mg mL^−1^ streptomycin unless otherwise stated.

Cell transfection was performed using lipofectamine 3000 or polyethylenimine (PEI), as described previously.^[^ ^70^, ^71^ ^]^ Packaging of lentiviral shRNA or cDNA expressing viruses, as well as subsequent infection of various cell lines were performed according to the protocols described previously.^[^ ^71^, ^72^ ^]^ Following viral infection, cells were maintained in the presence of hygromycin (200 µg mL^−1^) or puromycin (2 µg mL^−1^), depending on the viral vector used to infect cells. Gene knockdown in shRNA‐tet‐on cells was achieved by refreshing medium containing 1 µg ml^−1^ of doxycycline (TCI chemicals, D4116) every 3 days.

### Plasmids

Flag‐STING‐WT, C88A, C91A, C88A/C91A were constructed by (overlap) PCR cloning *STING* into pCDNA3.0 vector using primer sets listed below. Flag‐STING‐HAQ‐H232R was constructed by PCR cloning HAQ‐H232R variant from THP‐1 cell cDNA into pCDNA3.0 vector. Flag‐STING‐H232R was constructed by PCR cloning H232R variant from hORFeome V5.1 collection into pCDNA3.0 vector. Flag‐STING‐HAQ‐Y199C‐H232R was constructed by overlapping PCR to clone HAQ‐H232R variant into pCDNA3.0 vector using primers listed below. pBabe‐Flag‐STING‐WT, C91A and C88A/C91A were constructed by PCR cloning WT, C91A and C88A/C91A into pBabe‐hygro vector. pLenti‐Flag‐STING‐WT, C88A/C91A and R238A were constructed by cloning WT, R238A and C88A/C91A into pLenti‐hygro vector. CMV‐GST‐STING‐WT and S366A were constructed by (overlap) PCR cloning WT and S366A into CMV‐GST vector. Flag‐STING‐1‐110, 1–150, 42–150, 82–150, 111–150, and 151–379 were constructed by PCR cloning respective *STING* truncations into CMV‐GST vector using primer sets listed below. Flag‐cGAS was as described previously.^[^ ^73^ ^]^ His‐Ub was as described previously.^[^ ^73^, ^74^ ^]^ HA‐VDAC2‐WT, C103A, C210A, C227A, C103A/C210A/C227A (3A), E84Q, Q101K, S128A, and L136F were constructed by (overlap) PCR cloning *VDAC2* into pCDNA3 vector using primer sets listed below. pLenti‐HA‐VDAC2 and ATP2A2 were constructed by PCR cloning *VDAC2* and *ATP2A2* into pLenti‐GFP‐hygro vector using primer sets listed below. CMV‐GST‐GRP75 was constructed by PCR cloning GRP75 from hORFeome V5.1 collection into CMV‐GST vector. CMV‐GST‐ZDHHC7‐WT and C160S were constructed by (overlap) PCR cloning *ZDHHC7* into CMV‐GST vector using primer sets listed below.

### Primers

Primers used in generating plasmids used in this study are listed below:

STING‐BamHI‐F: GACACCGACTCTAGAGGATCCATGCCCCACTCCAGCCTGCA
STING‐SalI‐Flag‐R: ATCCAGAGGTTGATTGTCGACTCACTTGTCGTCATCGTCTTTGTAGTC AGAGAAATCCGTGCGGAGAG
STING‐C88A‐F: TGGAGGACTGTGCGGGCCGCCCTGGGCTGCCCCCTCCG
STING‐C88A‐R: CGGAGGGGGCAGCCCAGGGCGGCCCGCACAGTCCTCCA
STING‐C91A‐F: TGCGGGCCTGCCTGGGCGCCCCCCTCCGCCGTGGGGCCC
STING‐C91A‐R: GGGCCCCACGGCGGAGGGGGGCGCCCAGGCAGGCCCGCA
STING‐C88A/C91A‐F: TGGAGGACTGTGCGGGCCGCCCTGGGCGCCCCCCTCCGCCGTGGGGCCC
STING‐C88A/C91A‐R: GGGCCCCACGGCGGAGGGGGGCGCCCAGGGCGGCCCGCACAGTCCTCCA
STING‐P141A‐F: CAAGGGCCTGGCCGCAGCTGAGATCTCTGCAGTG
STING‐P141A‐R: CACTGCAGAGATCTCAGCTGCGGCCAGGCCCTTG
STING‐Y199C‐F: AGTGAGCCAGCGGCTGTGTATTCTCCTCCCATTGG
STING‐Y199C‐R: CCAATGGGAGGAGAATACACAGCCGCTGGCTCACT
STING‐R238A‐F: GCTGGCATCAAGGATGCGGTTTACAGCAACAGCATCTATGAGC
STING‐R238A‐R: GCTCATAGATGCTGTTGCTGTAAACCGCATCCTTGATGCCAGC
STING‐S366A‐F: AGCCTGAGCTCCTCATCGCTGGAATGGAAAAGCCCCT
STING‐S366A‐R: AGGGGCTTTTCCATTCCAGCGATGAGGAGCTCAGGCT
STING‐42‐BamHI‐F: GCAT GGATCCATGCACACTCTCCGGTACCTGGT
STING‐82‐BamHI‐F: GCAT GGATCCATGTGGAGGACTGTGCGGGCCTG
STING‐111‐BamHI‐F: GCAT GGATCCATGAATGCGGTCGGCCCGCCCTT
STING‐110‐SalI‐Flag‐R: GCATGTCGACTCACTTGTCGTCATCGTCTTTGTAGTCTGGGAGGGAGTAGTAGAAAT
STING‐1/150‐SalI‐Flag‐R: GCATGTCGACTCACTTGTCGTCATCGTCTTTGTAGTCTTTTTCACACACTGCAGAGA
STING‐151‐BamHI‐F: GCATGGATCCATGGGGAATTTCAACGTGGCCCA
VDAC2‐HA‐EcoRI‐F (for pCDNA3):
GCATGAATTCATGTATCCATATGATGTTCCAGATTATGCTGCGACCCACGGACAGACTTG
VDAC2‐HA‐XbaI‐F (for pLenti‐GFP hygro):
GCATTCTAGAATGTATCCATATGATGTTCCAGATTATGCTGCGACCCACGGACAGACTTG
VDAC2‐SalI‐R: GCAT GTCGAC TTAAGCCTCCAACTCCAGGG
VDAC2‐C103A‐F: CAATTGAAGACCAGATTGCTCAAGGTTTGAAACTGA
VDAC2‐C103A‐R: TCAGTTTCAAACCTTGAGCAATCTGGTCTTCAATTG
VDAC2‐C210A‐F: CAATTTATCAGAAAGTTGCTGAAGATCTTGACACTT
VDAC2‐C210A‐R: AAGTGTCAAGATCTTCAGCAACTTTCTGATAAATTG
VDAC2‐C227A‐F: GGACATCAGGTACCAACGCCACTCGTTTTGGCATTG
VDAC2‐C227A‐R: CAATGCCAAAACGAGTGGCGTTGGTACCTGATGTCC
VDAC2‐E84Q‐F: GAGTATGGTCTGACTTTCACACAAAAGTGGAACACTGATAACACTCTG
VDAC2‐E84Q‐R: CAGAGTGTTATCAGTGTTCCACTTTTGTGTGAAAGTCAGACCATACTC
VDAC2‐Q101K‐F: CAGAAATCGCAATTGAAGACAAGATTTGTCAAGGTTTGAAACTGAC
VDAC2‐Q101K‐R: GTCAGTTTCAAACCTTGACAAATCTTGTCTTCAATTGCGATTTCTG
VDAC2‐S128A‐F: GAAAAGTGGTAAAATCAAGTCTGCTTACAAGAGGGAGTGTATAAACC
VDAC2‐S128A‐R: GGTTTATACACTCCCTCTTGTAAGCAGACTTGATTTTACCACTTTTC
VDAC2‐L136F‐F: CAAGAGGGAGTGTATAAACTTTGGTTGTGATGTTGACTTTGATTTTGC
VDAC2‐L136F‐R: GCAAAATCAAAGTCAACATCACAACCAAAGTTTATACACTCCCTCTTG
ATP2A2‐BglII‐HA‐F: GCATAGATCTATGTATCCATATGATGTTCCAGATTATGCTGAGAACGCGCACACCAAGAC
ATP2A2‐Flag‐SalI‐R: GCATGTCGACTCACTTGTCGTCATCGTCTTTGTAGTCAGACCAGAACATATCGCTAA
GRP75‐BamHI‐F: GCAT GGATCC ATGATAAGTGCCAGCCGAGC
GRP75‐SalI‐R: GCAT GTCGAC TTACTGTTTTTCCTCCTTTTG
ZDHHC7‐BamHI‐F: GCATGGATCCATGCAGCCATCAGGACACAG
ZDHHC7‐SalI‐R: GCAT GTCGAC TCACACTGAGAACTCCGGGC
ZDHHC7‐sh3‐resist‐F: AGTGATTTTTCACCTCCCATTACAGTAATCCTGTTGATCTT
ZDHHC7‐sh3‐resist‐R: AAGATCAACAGGATTACTGTAATGGGAGGTGAAAAATCACT
ZDHHC7‐C160S‐F: GAAAATGGATCATCACTCCCCGTGGGTGAACAATT
ZDHHC7‐C160S‐R: AATTGTTCACCCACGGGGAGTGATGATCCATTTTC
RT‐PCR primers are listed below:
STING1‐F: CACTTGGATGCTTGCCCTC
STING1‐R: GCCACGTTGAAATTCCCTTTTT
HMOX1‐F: AAGACTGCGTTCCTGCTCAAC
HMOX1‐R: AAAGCCCTACAGCAACTGTCG
CYB5R3‐F: TCTACCTCTCGGCTCGAATTG
CYB5R3‐R: CCTTGTCATCATCGCTGGAGAT
TXNIP‐qPCR‐F: ATATGGGTGTGTAGACTACTGGG
TXNIP‐qPCR‐R: GACATCCACCAGATCCACTACT
ZDHHC3‐F: CCACTTCCGAAACATTGAGCG
ZDHHC3‐R: CCACAGCCGTCACGGATAAA
ZDHHC7‐F: CCCAAAGGAAACGCTACGAAA
ZDHHC7‐R: CGCGCTCGGGTTTAATACAG
RNA18S‐F: TGCGGAAGGATCATTAACGGA
RNA18S‐R: AGTAGGAGAGGAGCGAGCGACC
U6‐qPCR‐F: CTCGCTTCGGCAGCACA
U6‐qPCR‐R: AACGCTTCACGAATTTGCGT

### shRNAs and sgRNAs

shRNA plasmids to deplete endogenous *STING*, *cGAS, ZDHHC3*, and *ZDHHC7* were purchased from Sigma. Their target sequence is listed below:

shScr: CCGGAACAGTCGCGTTTGCGACTGGCTCGAGCCAGTCGCAAACGCGACTGTTTTTTTTG
shSTING‐1: CCGGCCAACATTCGCTTCCTGGATACTCGAGTATCCAGGAAGCGAATGTTGGTTTTTTG
shSTING‐2: CCGGGCAGAGCTATTTCCTTCCACACTCGAGTGTGGAAGGAAATAGCTCTGCTTTTTTG
shSTING‐3: CCGGGTCCAGGACTTGACATCTTAACTCGAGTTAAGATGTCAAGTCCTGGACTTTTTTG
shcGAS‐1: CCGGCGTGAAGATTTCTGCACCTAACTCGAGTTAGGTGCAGAAATCTTCACGTTTTTTG
shcGAS‐2: CCGGATCTATTCTCTAGCAACTTAACTCGAGTTAAGTTGCTAGAGAATAGATTTTTTG
shZDHHC3‐10: CCGGGTATAGCATCATCAACGGAATCTCGAGATTCCGTTGATGATGCTATACTTTTTTG
shZDHHC3‐18: CCGGGCTTTGAAGAAGATTGGACAACTCGAGTTGTCCAATCTTCTTCAAAGCTTTTTTG
shZDHHC3‐79: CCGGCCAGAAGTACTTCGTCCTGTTCTCGAGAACAGGACGAAGTACTTCTGGTTTTTTG
shZDHHC7‐3: CCGGGATAACTGTAATCCTGTTGATCTCGAGATCAACAGGATTACAGTTATCTTTTTTG
shZDHHC7‐34: CCGGACTGCCCGTGGGTGAACAATTCTCGAGAATTGTTCACCCACGGGCAGTTTTTTTG

Other shRNA plasmids were constructed by inserting synthesized shRNAs into pLKO‐puro or pLKO‐hydro vectors. Their target sequence is listed below:

shMCU: CCGGGCAAGGAGTTTCTTTCTCTTTCTCGAGAAAGAGAAAGAAACTCCTTGCTTTTTG
shHIF‐1*α*: CCGGCCAGTTATGATTGTGAAGTTACTCGAGTAACTTCACAATCATAACTGGTTTTTTG
shHIF‐2*α*: CCGGGGAGACGGAGGTGTTCTATTTCAAGAGAATAGAACACCTCCGTCTCCTTTTTTG
shGRP75‐51:
CCGGGCACATTGTGAAGGAGTTCAACTCGAGTTGAACTCCTTCACAATGTGCTTTTTTG
shGRP75‐52:

Tet‐inducible shRNA plasmids were constructed by inserting synthesized shRNAs into Tet‐pLKO‐neo vector. Target sequence of STING was the same as that of shSTING‐2 and ‐3. Target sequence of shVDAC2 is listed below:

shVDAC2: CCGGAAGGATGATCTCAACAAGAGCCTCGAGGCTCTTGTTGAGATCATCCTTTTTTTTG

sgRNA plasmids were constructed by inserting synthesized sgRNAs into lentiCRISPRv2‐puro vector. Their target sequence is listed below:

sgSTING‐1B: GCTGGGACTGCTGTTAAACG
sgSTING‐6: CATTACAACAACCTGCTACG
sgcGAS‐1: CACGTGCTCATAGTAGCTCC
sgcGAS‐2: GGCCGCCCGTCCGCGCAACT
sgIRF3‐1: CGCTCACTGCCCAGTATGTG
sgIRF3‐4: GGCACCAACAGCCGCTTCAG
sgMCU: GTGTTTTCTAGGTACACCAG

STING‐S366A knockin experiment was performed using STING sgRNAs and ssoDNA as listed below:

STING‐S366A‐KI‐sgF: CACCGGCTTTTCCATTCCACTGATG
STING‐S366A‐KI‐sgR: AAACCATCAGTGGAATGGAAAAGCC
STING‐S366A‐KI‐ssODNA
GGGCAGCTTGAAGACCTCAGCGGTGCCCAGTACCTCCACGATGTCCCAAGAGCCTGAGCTATTAATCGCTGGAATGGAAAAGCCCCTCCCTCTCCGCACGGATTTCTCTTGAGACCCAGGGTCACCAGGCCAGAGCCTCC

Knockin clones were screened by PCR using primers listed below to search for clones loss of SacI but gain of AseI site after knockin.

STING‐S366A‐KI‐PCR‐F: GAGTGGGAATGGGTAAGATCCTC
STING‐S366A‐KI‐PCR‐R: GACGCATCTTAAGATGTCAAGTCC

### Immunoblot and Immunoprecipitations Analyses

Cells were lysed in EBC buffer (50 mM Tris pH 7.5, 120 mM NaCl, 0.5% NP‐40) or Triton X‐100 buffer (50 mM Tris pH 7.5, 150 mM NaCl, 1% Triton X‐100) supplemented with protease inhibitor cocktail (Bimake, B14002) and phosphatase inhibitor cocktail (Bimake, B15002). The protein concentrations of whole cell lysates were measured by NanoDrop OneC using the Bio‐Rad protein assay reagent as described previously.^[^ ^70^, ^71^ ^]^ Equal amounts of whole cell lysates were resolved by SDS‐PAGE and immunoblotted with indicated antibodies. For immunoprecipitations analysis, unless specified, 1 mg lysates were incubated with the indicated antibody (1–2 µg) for 3–4 h at 4 °C followed by 1 h incubation with 10 µL Protein A/G XPure Agarose Resin (UBPBio, P5030‐5). Or 1 mg lysates containing tagged molecules were incubated with agarose beads coupled antibodies for the specific tag for 3–4 h at 4 °C. For endogenous IPs, incubation of cell lysates with antibodies was extended to overnight. The recovered immuno‐complexes were washed five times with NETN buffer (20 mM Tris, pH 8.0, 100 mM NaCl, 1 mM EDTA, and 0.5% NP‐40) before being resolved by SDS‐PAGE and immunoblotted with indicated antibodies. For VDAC2 oligomerization detection, cells were incubated with 250 µM ethylene glycol bis (succinimidyl succinate) (Fisher Scientific, 70539‐42‐3), lyzed in Triton X‐100 buffer and nixed with 4 × lithium dodecyl sulfate sample buffer (ThermoFisher Scientific, NP0007). Load protein without boiling in SDS‐free PAGE gel and immunoblotted with indicated antibodies.

### Generation of STING‐S366A Knockin A498 Cells

Parental A498 cells were split into 24‐well plates and transfected with sgRNA against endogenous STING together with STING‐S366A‐ssoDNA following protocols as described.^[^ ^60^ ^]^ 1‐day post‐transfection, cells were selected with 1 µg ml^−1^ puromycin for 3 days. Surviving cells were counted and each single cell was seeded into 96‐well plates. Each single clone grown up in 96‐well plates was amplified and one copy was used for genomic DNA extraction, followed by PCR and AseI/SacI digestion to screen for potential knockin clones. SacI negative but AseI positive clones are selected and sequenced to verify the knockin at the DNA level.

### Mass Spectrometry, Data Filtering, and Bioinformatics

A498 cells stably expressing either FLAG‐EV or FLAG‐STING were lysed in EBC lysis buffer FLAG immunoprecipitants were washed with NETN buffer. Proteins were diluted in 8 m urea (Sigma; U4883) to 100 µg µl^−1^ and subjected to FASP trypsin digestion protocol. Briefly, proteins were reduced using 50 mM DTT (Pierce; A39255) for 15 min at 65 °C. Proteins were then transferred to a 30 000 MWCO spin filter (Vivacon 500; 14‐558‐349), diluted with 200 µl of 8 m urea, and centrifuged at 10 000 × g for 30 min at RT. The proteins were washed twice using 200 µl of 8 m urea followed by 30 minutes of centrifugation at 10 000 × g between each wash. Proteins were alkylated using 100 µl of 15 mM 2‐chloroacetamide (Acros Organics; 148415000) prepared in 8 m urea for 20 min in the dark at RT. The spin filter was then centrifuged at 10 000 × g for 20 min at RT followed by two washes with 200 µl of 8 M urea with 20 min of centrifugation at 10000 × g in between each wash. Buffer exchange into 50 mM ammonium bicarbonate (ABC) pH 8.0 was performed with two washes of 200 µl of 50 mM ABC with centrifugation at 10 000 × g for 15 min at RT between each wash. 100 µl of 50 mM ABC was added to the spin filter along with 2.5 µg of trypsin (Promega V511C). Proteins were trypsinized overnight at 37°C for 18 h. Following trypsinization, peptides were recovered in a new receiver tube by centrifugation at 10 000 × g for 15 min. Peptides were eluted twice using 50 µl of 0.5% TFA in water at 10 000 × g for 10 min. Samples were then concentrated to 100 µl using a speedvac followed by C18 desalting (ThermoScientific; 89870). Samples were then concentrated using a speedvac and resolubilized in 100 µl of LC‐Optima MS‐grade water (Thermo). Ethyl acetate extraction followed by speedvac was performed to remove residual detergents. QFP assay (Thermo; 23290) was performed for peptide quantification.

Detailed liquid chromatography‐mass spectrometry/mass spectrometry methods and data filtering methods were described previously.^[^ ^75^, ^76^ ^]^ Data was searched using MaxQuant (version 1.6.6.7), and all statistical analyses were done in Perseus (version 1.6.3.4).

### RNA Extraction and qRT‐PCR

RNA extraction was performed with an RNA miniprep super kit (BioBasic, BS584) and QIAshredder (Qiagen, 79654). The final elution step was done with 50 µL of RNAse‐free water. The relative enrichment of mRNA was quantified with the NanoDrop OneC (ThermoFisher Scientific). At least two biological replicates were performed for RNA extraction. Reverse transcription was performed with iScript cDNA synthesis kit (BIO‐RAD, 1708891). Quantitative real‐time PCR was performed with iTaq universal SYBR green supermix (BIO‐RAD, 1725124) using a QuantStudio 6 Flex Real‐Time PCR Systems (ThermoFisher Scientific). Each mRNA level was normalized to RNA18S or U6 snRNA. The comparative Ct method was used to calculate fold change in expression. Statistical significance was determined by one‐way ANOVA tests.

### Cell Viability Assays

Indicated number of cells were seeded in each well of 96‐well plates for cell viability assay to monitor cell viability at indicated time periods using Cell Counting Kit 8 (CCK8, Bimake, B34304) according to manufacturer's instruction. Briefly, at indicated time points post‐cell seeding, 10 µL CCK8 solution was added into each well and incubated in the culture incubator (37 °C with 5% CO_2_) for 3 h. After thorough mixing, absorbance at 450 nm was measured using the BioTek Cytation 5 Cell Imaging reader. Combination index (CI) of co‐treatment of 2‐BP and sorafenib was calculated by CompuSyn software (ComboSyn, Inc).

### Colony Formation Assays

Indicated cells were seeded into 6‐well or 24‐well plates (500 cells/well) and cultured in 37 °C incubator with 5% CO_2_ for 7–15 days (as indicated in figure legends) until formation of visible colonies. Colonies were washed with 1x PBS, fixed with methanol for 30 min, and stained with 0.5% crystal violet for 30 min. Colonies were then washed with distilled water and air‐dried. Colony numbers were manually counted. Three independent experiments were performed to generate the error bars.

### Soft Agar Assays

The anchorage‐independent cell growth assays were performed as described previously.^[^ ^53^ ^]^ Briefly, the assays were preformed using 6‐well plates where the solid medium consists of two layers. The bottom layer contains 0.8% noble agar, and the top layer contains 0.4% agar suspended with 3 × 10^4^ or indicated number of cells. 500 µL complete DMEM medium with 10% FBS was added every 4 days. About 4–6 weeks later the cells were stained with iodonitrotetrazolium chloride (1 mg mL^−1^) (Sigma I10406) overnight for colony visualization, imaging, and counting. Three independent experiments were performed to generate the error bar.

### Mouse Xenograft Assays

All mouse work has been reviewed and approved by UNC Institutional Animal Care and Use Committee under IACUC#19‐031 which has been continued as #22‐056. Mouse xenograft assays were performed as described previously.^[^ ^70^, ^71^ ^]^ Briefly, for mouse xenograft growth experiments, A498 cells were infected with viruses expressing shScr or shSTING2/3. Two days later, 1 × 10^6^ A498 cells in PBS were injected into the flank of indicated female nude mice (NCRNU‐M‐M from UNC Animal Facility, 4 weeks old). Tumor size was measured every two days with a digital caliper, and the tumor volume was determined with the formula: *L* × *W* ^2^ × 0.5, where *L* is the longest diameter and *W* is the shortest diameter. After 66 days, mice were sacrificed, and tumors were dissected and weighed. For inducible STING shRNA, 1 × 10^7^ A498 cells in DMEM were injected into flank of male nude mice (Jackson Laboratory, 5 weeks old). When tumors became visible after 15 days, 2 mg ml^−1^ doxycycline was added to drinking water containing 2% sucrose. Water was changed every three days and protected from light. Mice were sacrificed 39 days after injection. For combination therapy, 1 × 10^7^ A498 cells in DMEM were injected into flank of male nude mice (Jackson Laboratory, 5 weeks old). After 25 days, when tumors reached a volume of ≈150 mm^3^, mice were randomly divided into four groups. 2‐BP was dissolved in 10% DMSO+40% PEG300+5% Tween 80+ 45% saline. Sorafenib was dissolved in 3% DMSO+97% sulfobutylether‐*β*‐Cyclodextrin solution (20% in saline). Mice were daily treated with 40 mg kg^−1^ 2‐BP given by intraperitoneal injection or 40 mg kg^−1^ sorafenib given by oral gavage or both drugs or their solvents. Tumor size was measured regularly, and the tumor volume was determined with the formula: *L* x *W* ^2^ x 0.5, Mice were sacrificed after 13 days of treatment.

### RNA‐Seq

Total RNA from triplicate samples of A498 cells infected with shScr or shSTING was extracted with RNeasy mini kit (Qiagen, 74104). Library preparation and sequencing were performed by GENEWIZ as paired‐end 150‐bp reads. Reads were then filtered for adaptor contamination using cutadapt and filtered such that at least 90% of bases of each read had a quality score > 30. Reads were aligned to the reference genome (hg19) using STAR version 2.5.2b retaining only primary alignments.^[^ ^77^ ^]^ Reads overlapping blacklisted regions of the genome were then removed. Transcript abundance was then estimated using Salmon,^[^ ^78^ ^]^ and differentially expressed genes were detected using DESeq2 with the criteria of adjusted *p*‐values (adjP) <0.05.^[^ ^79^ ^]^ The ClusterProfiler R package (v3.14.3) was employed to analyze the Gene Ontology (GO) and Kyoto Encyclopedia of Genes and Genomes (KEGG) for functional pathway annotation. Enrichment analysis for GO terms and KEGG pathways utilize enrichGO and enrichKEGG functions and visualizes the result with bubble plots. RNA‐seq data are deposited to GEO under accession number GSE190816.

### FACS Analyses

To measure cell cycle phase distribution, cells were treated with 10 µM EdU (Santa Cruz Biotechnology, sc‐284628) for 30 min. before harvesting by trypsinization. Cells were washed with PBS and then fixed in 4% paraformaldehyde in PBS for 15 min. at room temperature. 1% BSA‐PBS was added, mixed, and cells were centrifuged. Fixed cells were permeabilized with 0.5% triton X‐100 in 1% BSA‐PBS at room temperature for 15 min. and centrifuged. Cells were then processed for EdU detection as follows: samples were incubated in PBS with 1 mM CuSO_4_, 1 µM Alexa 647‐azide (Life Technologies), and 100 mM ascorbic acid (fresh) for 30 min. at room temperature in the dark. Finally, cells were resuspended in 1% BSA‐PBS with 1 µg mL^−1^ DAPI (Life Technologies) and 100 µg mL^−1^ RNAse A (Sigma Aldrich) and incubated overnight at 4 °C in the dark. Data were collected on an Attune NxT flow cytometer (Thermo Fisher Scientific) and analyzed using FCS Express 7 software (De Novo Software).

To measure general ROS level, trypsinized cells were incubated with 10 µM CM‐H2DCFDA (ThermoFisher Scientific, C6827) in PBS at 37 °C for 30 min. To measure mitochondrial ROS level, trypsinized cells were incubated with 1 µM MitoSOX Red mitochondrial superoxide indicator (ThermoFisher Scientific, M36008) in PBS at 37 °C for 20 min. To measure mitochondrial calcium level, trypsinized (without EDTA) cells were incubated with 5 µM Rhod‐2, AM (ThermoFisher Scientific, R1244) in PBS solution containing 10% FBS and 10 mM glucose at 37 °C for 1 h. To measure mitochondrial membrane potential, cells were incubated with 2 µM JC‐1 (AdipoGen, AG‐CR1‐3568) in PBS at 37 °C for 15 min. Cells were washed with PBS twice and analyzed with BD FACScanto II or BD FACSfortessa (BD Biosciences) with the BD Diva software (BD Biosciences). A minimum of 10 000 events were acquired for each sample. Data were analyzed using Flowjo 7.6.1 (Tree Star).

### Immunofluorescence

Cells plated onto glass coverslips were fixed with 4% paraformaldehyde in PBS for 20 min at room temperature and permeabilized with 0.2% Triton X‐100 for 20 min at room temperature. Cells were incubated with blocking buffer (5% bovine serum albumin and 0.1% Triton X‐100 in PBS) for 1 h, incubated with primary antibodies at 4 °C overnight, incubated with secondary antibodies and/or mitochondria dye MitoView Green (Biotium, 70054) at room temperature for 1 h and mounted with ProLong Gold antifade reagent (Invitrogen, P36931). Fluorescent signals were observed with an Olympus 1 × 51 inverted microscope at × 20 magnification, or with an Olympus FV1000 confocal microscope at 60× magnification.

### Mitochondria Isolation

Cytosolic and mitochondrial fractions of cells were isolated with Mitochondria isolation kit (Thermo Scientific, 89874) for cultured cells according to manufacturer's instruction. Briefly, add 800 µl reagent A to cell pellets and incubate for 2 min. Added 10 µl reagent B and vortex 5 s min^−1^. Added 800 µl reagent C and invert tubes several times to mix. Centrifuged tubes at 700 × g for 10 min and collect supernatant as a mixture containing mitochondria, lysosome, and peroxisome. Centrifuged supernatant at 3000 × g for 15 min and this pellet contains isolated mitochondria. Centrifuged supernatant at 12 000 × g for 15 min and transferred new supernatant containing cytosolic fraction to a new tube. The pellet was a mixture of lysosome, peroxisome, and some mitochondria. Resuspended mitochondria pellet in 500 µl reagent C and centrifuged at 12 000 × g for 5 min to obtain pure mitochondrial fraction. Samples were kept at 4 °C throughout the procedure.

### Electron Microscopy

Electron microscopic analysis of indicated A498 cells was performed as detailed below. Sample prep: indicated A498 cell culture pellets were embedded in 2% agarose, secondary fixation with osmium tetroxide, dehydration (25,50,70,95100% EtOH), followed by embedding in EPON. Sectioning: ≈80 nm thin sections on Cu grids, stained with UA and LC. Imaging: FEI Tecnai 12 at 120 kV, Gatan Rio16 CMOS camera. Cells were randomly selected for imaging. Images at 4400× magnification were used for counting total and abnormal mitochondria. All clearly identified mitochondria in the images were analyzed. Images at 26 000× magnification were randomly selected for mitochondria‐ER contact (MERC) length measurement. All clearly identified mitochondria membranes and associated ER membranes in the images were evaluated. Only MERC sites with a distance no more than 30 nm were regarded as MERC enabling calcium transfer from ER to mitochondria and their length was measured according to.^[^ ^80^ ^]^ Image analysis was performed using Adobe Illustrator.

### OCR and ECAR Measurement

The OCR and ECAR were measured by an XFe24 extracellular flux analyzer (Agilent Technologies), according to the manufacturer's instructions. Briefly, 5 × 10^4^ cells were seeded into XF24 cell culture microplate (Agilent Technologies, 100777‐004) before the assay. Added 1 mL of Seahorse XF24 calibrant solution (pH 7.4) (Agilent Technologies, 100840–100) to each well of XF24 microplate and replaced green sensor cartridge on the top. Incubated entire cartridge in a non‐CO_2_ incubator at 37°C overnight. On the day following cell seeding, media was changed to phenol red‐free, Seahorse XF DMEM medium, pH 7.4 (Agilent Technologies, 103575‐100) supplemented with 25 mM glucose and 1 mM sodium pyruvate for OCR or 2 mM glutamine and 1 mM sodium pyruvate for ECAR. Cells were equilibrated within 1 h in 37 °C non‐CO_2_ incubator. Loaded the sensor cartridge for the calibration process and replaced calibration plate with cell plate after that. The basal mitochondrial respiration or extracellular acidification rate was first measured by recording extracellular oxygen concentration. Then the OCR or ECAR trace was recorded in response to sequential addition of indicated compounds in the Seahorse XF Cell Mito Stress Test Kit (Agilent Technologies, 103015–100) or Glycolysis Stress Test Kit (Agilent Technologies, 103020–100). Concentration of compounds for OCR was: 1 µM of oligomycin A, 1 µM of FCCP, and 1 µM of rotenone/antimycin A. Concentration of compounds for ECAR was: 10 mM of glucose, 1 µM of oligomycin, and 50 mM of 2‐DG. 3 to 5 technical replicates were utilized per sample to calculate OCR or ECAR.

### STING Protein Palmitoylation Detection

Protein palmitoylation detection was performed by immunoprecipitation and acyl‐biotin exchange as described previously.^[^ ^81^ ^]^ Briefly, cells were lysed in lysis buffer (LB) (1% NP‐40, 50 mM Tris pH 7.5, 150 mM NaCl, 10% Glycerol) supplemented with protease inhibitor, PMSF and 50 mM *N*‐ethylmaleimide (NEM) (Acros Organics, 156100100). Equal amounts of lysates were incubated with the STING antibody overnight at 4 °C followed by 1 h incubation with 60 µL Protein A/G XPure Agarose Resin, or incubated with Flag agarose beads for 3–4 h at 4 °C. Beads were resuspended in LB+10 mM NEM and split into triplicates. 1/3 of beads were used as ‐HAM control and 2/3 were used as +HAM treatment. Beads were resuspended with LB (pH 7.2)+0.1% SDS quickly, washed with LB (pH = 7.2) three times, resuspended in LB (pH 7.2) with or without 1 m hydroxylamine (HAM) (Thermo Scientific, A15398.30) and incubated at room temperature for 1 h. Then, beads were washed with LB (pH 6.2) once and resuspended in LB (pH = 6.2)+2 µM Biotin‐BMCC (Thermo Scientific, 21900) at 4 °C for 1 h. Beads were then washed with LB (pH = 6.2) once and LB (pH 7.5) for three times. Beads were boiled and resolved by SDS‐PAGE and immunoblotted with streptavidin‐HRP antibody to detect biotin‐labeled palmitoylated STING.

### Statistical Analysis

Statistical analyses were performed using the Graphpad Prism 8 Software. *p* ≤ 0.05 was considered statistically significant. The results were shown as means ± SD from at least two or three independent experiments as indicated in figure legends. Differences between control and experimental conditions were evaluated by One‐way ANOVA.

## Conflict of Interest

The authors declare no conflict of interest.

## Author Contributions

Conceptualization: ZZ, PL; Methodology: ZZ, JGC, BM, XT, HY, GD, PL; Investigation: ZZ, HD, XZ, EWC, JZ, LM, YW, XT, TS, JS; Visualization: ZZ, YW, JZ; Funding acquisition: JGC, DH, GD, PL; Project administration: JLS, EH; Supervision: JGC, BM, GD, PL; Writing – original draft: ZZ, PL; Writing – review & editing: HY, JGC, BM, GD.

## Supporting information

Supporting Information

## Data Availability

The data that support the findings of this study are available from the corresponding author upon reasonable request.
