# Supplementary material for: STING Suppresses Mitochondrial VDAC2 to Govern RCC Growth Independent of Innate Immunity
Source: Adv Sci (Weinh). 2022 Nov 29;10(3):2203718. doi: 10.1002/advs.202203718 (PMC9875608; doi:10.1002/advs.202203718)
Supplement: Supplementary file 1 — Supporting Information [file ADVS-10-2203718-s001.pdf]

ADVANCED SCIENCE
Open Access

Supporting Information

for *Adv. Sci.*, DOI 10.1002/advs.202203718

STING Suppresses Mitochondrial VDAC2 to Govern RCC Growth Independent of Innate Immunity

*Zhichuan Zhu, Xin Zhou, Hongwei Du, Erica W. Cloer, Jiaming Zhang, Liu Mei, Ying Wang, Xianming Tan, Austin J. Hepperla, Jeremy M. Simon, Jeanette Gowen Cook, Michael B. Major, Gianpietro Dotti and Pengda Liu**

ADVANCED SCIENCE
Open Access

Supporting Information for

STING suppresses mitochondrial VDAC2 to govern RCC growth independent of innate immunity

Zhichuan Zhu, Xin Zhou, Hongwei Du, Erica W. Cloer, Jiaming Zhang, Liu Mei, Ying Wang, Xianming Tan, Austin J. Hepperla, Jeremy M. Simon, Jeanette Gowen Cook, Michael B. Major, Gianpietro Dotti and Pengda Liu

correspondence to: pengda_liu@med.unc.edu

This PDF file includes:

Figs. S1 to S9

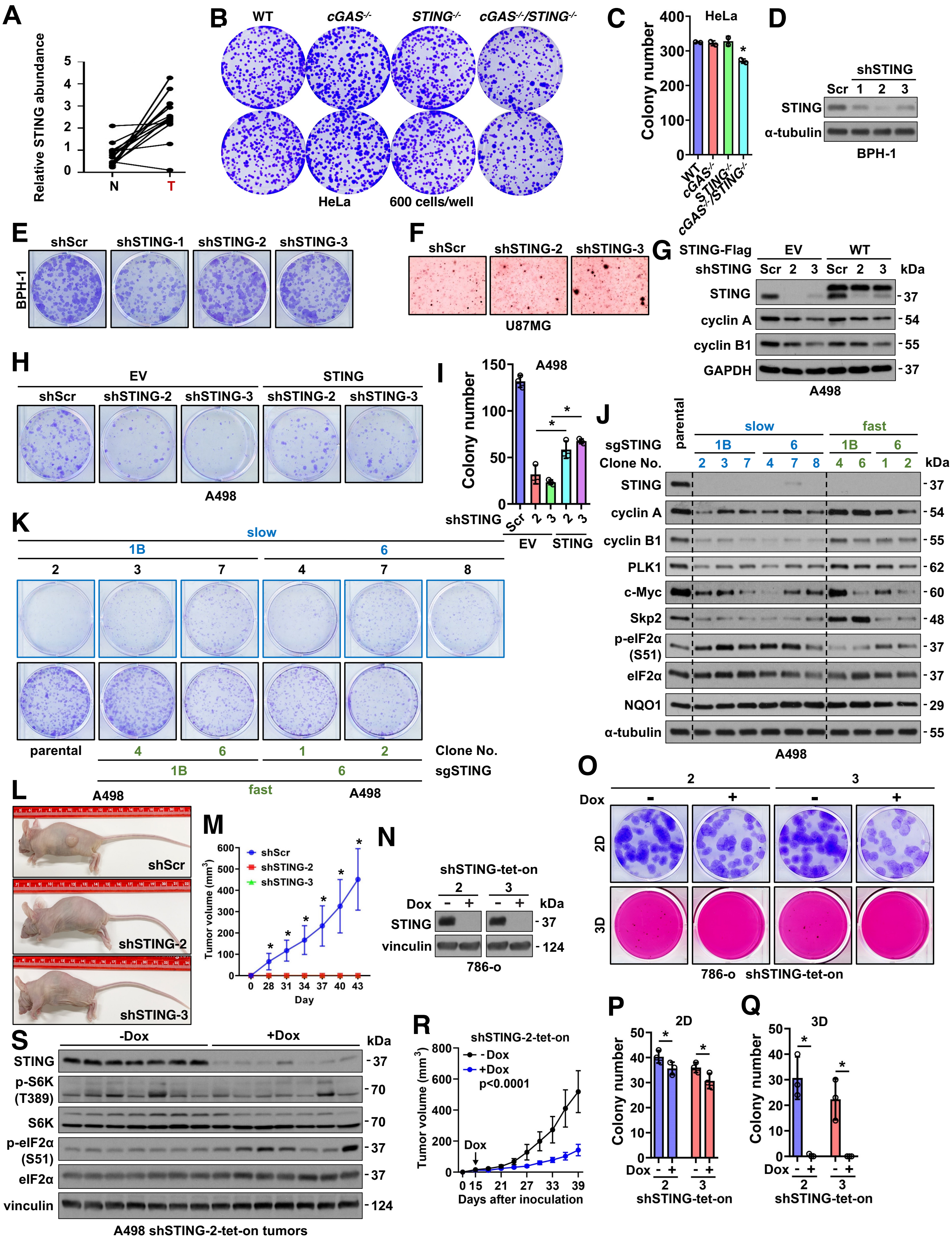
A
Relative STING abundance
N
T
B
WT
cGAS-/-
STING-/-
cGAS-/-/STING-/-
HeLa
600 cells/well
C
HeLa
Colony number
WT
cGAS-/-
STING-/-
cGAS-/-/STING-/-
D
shSTING
Scr 1 2 3
STING
α-tubulin
BPH-1
E
shScr
shSTING-1
shSTING-2
shSTING-3
BPH-1
F
shScr
shSTING-2
shSTING-3
U87MG
G
STING-Flag
EV
WT
shSTING Scr 2 3 Scr 2 3
kDa
STING
cyclin A
cyclin B1
GAPDH
A498
H
EV
STING
shScr
shSTING-2
shSTING-3
shSTING-2
shSTING-3
A498
I
A498
Colony number
shSTING
Scr 2 3 2 3
EV
STING
J
parental
slow
fast
sgSTING
1B
6
1B
6
Clone No.
2 3 7 4 7 8 4 6 1 2
kDa
STING
cyclin A
cyclin B1
PLK1
c-Myc
Skp2
p-eIF2α (S51)
eIF2α
NQO1
α-tubulin
A498
K
slow
1B
6
2 3 7 4 7 8
parental
4 6 1 2
Clone No.
1B
6
sgSTING
fast
A498
L
A498
shScr
shSTING-2
shSTING-3
M
shScr
shSTING-2
shSTING-3
Tumor volume (mm3)
Day
N
shSTING-tet-on
2 3
Dox - + - +
kDa
STING
vinculin
786-o
O
2
3
Dox - + - +
2D
3D
786-o shSTING-tet-on
P
2D
Colony number
Dox - + - +
2 3
shSTING-tet-on
Q
3D
Colony number
Dox - + - +
2 3
shSTING-tet-on
R
shSTING-2-tet-on
-Dox
+Dox
p<0.0001
Tumor volume (mm3)
Dox
Days after inoculation
S
-Dox
+Dox
kDa
STING
p-S6K (T389)
S6K
p-eIF2α (S51)
eIF2α
vinculin
A498 shSTING-2-tet-on tumors

Figure S1. STING depletion reduces RCC cell growth. (A) A quantification of Fig. 1C. (B) Representative images for 2D colony formation assays using indicated HeLa cells and quantified in (C). Error bars were calculated as mean+/-SD, n=3. *p < 0.05 (one-way *ANOVA* test). (D) IB analyses of WCL from indicated BPH-1 cells. (E) Representative images for 2D colony formation assays using BPH-1 cells obtained in (D). (F) Representative images for 3D sift agar growth assays using indicated U87MG cells. (G) IB analyses of WCL from indicated A498 cells. Where indicated, WT-STING was stably expressed in A498 cells by lentiviral infection, followed by infection with control or STING shRNA containing viruses to deplete endogenous STING. (H) Representative images for 2D colony formation assays using A498 cells obtained in (G) and quantified in (I). Error bars were calculated as mean+/-SD, n=3. *p < 0.05 (one-way *ANOVA* test). (J) IB analyses of WCL from indicated A498 single clones from infection with sgRNAs against endogenous STING. (K) Representative images for 2D colony formation assays using A498 cells obtained in (J). (L) Representative images for nude mice injected with indicated A498 cells. (M) Tumor volume measurements at indicated days post-injection of indicated A498 cells. Error bars were calculated as mean+/-SD, n=8. *p < 0.05 (one-way *ANOVA* test). (N) IB analyses of WCL from A498 or RCC10 cells depleted of endogenous cGAS. (O) Representative images of 2D colony formation assays using cells obtained in (N) and quantified in (P and Q). Error bars were calculated as mean+/-SD, n=3. *p < 0.05 (one-way *ANOVA* test). (R) Tumor volume measurements at indicated days post-injection of indicated A498 cells. Error bars were calculated as mean+/-SD, n=8. *p < 0.05 (one-way *ANOVA* test). (S) IB analysis of WCL derived from indicated A498 tumors from (R).

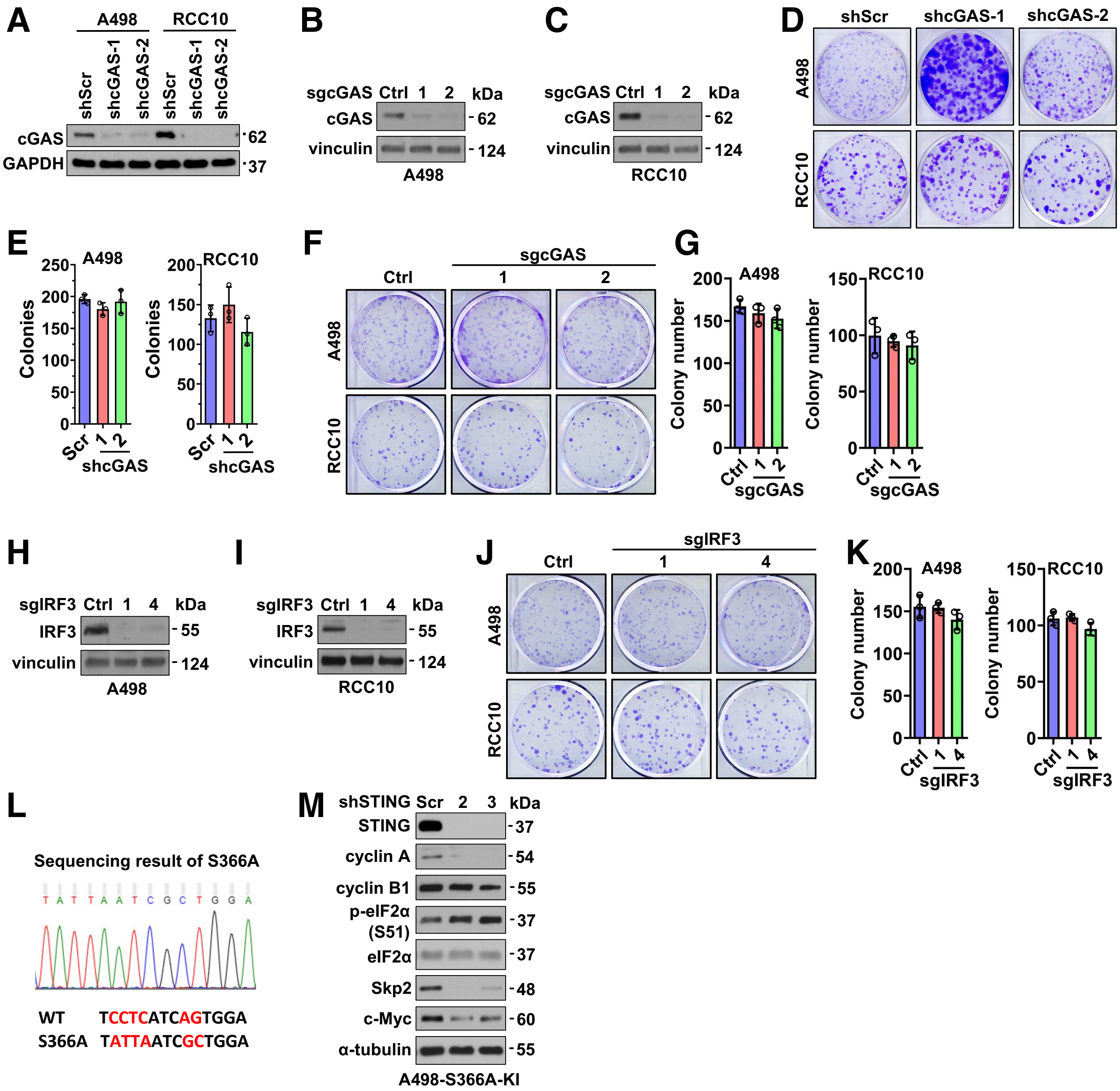

Figure S2. STING depletion reduces RCC cell growth independent of STING's innate immunity function. (A, B, C) IB analysis of WCL derived from indicated cGAS depleted A498 and RCC10 cells. (D, F) Representative images of 2D colony formation assays using cells obtained in (A-C) and quantified in (E, G). Error bars were calculated as mean+/-SD, n=3. *p < 0.05 (one-way *ANOVA* test). (H, I) IB analysis of WCL derived from indicated cGAS depleted A498 and RCC10 cells. (J) Representative images of 2D colony formation assays using cells obtained in (H, I) and quantified in (K). Error bars were calculated as mean+/-SD, n=3. *p < 0.05 (one-way *ANOVA* test). (L) DNA sanger sequencing result to validate the STING-S366A knockin A498 clones. (M) IB analysis of WCL from indicated A498-S366A knockin cells.

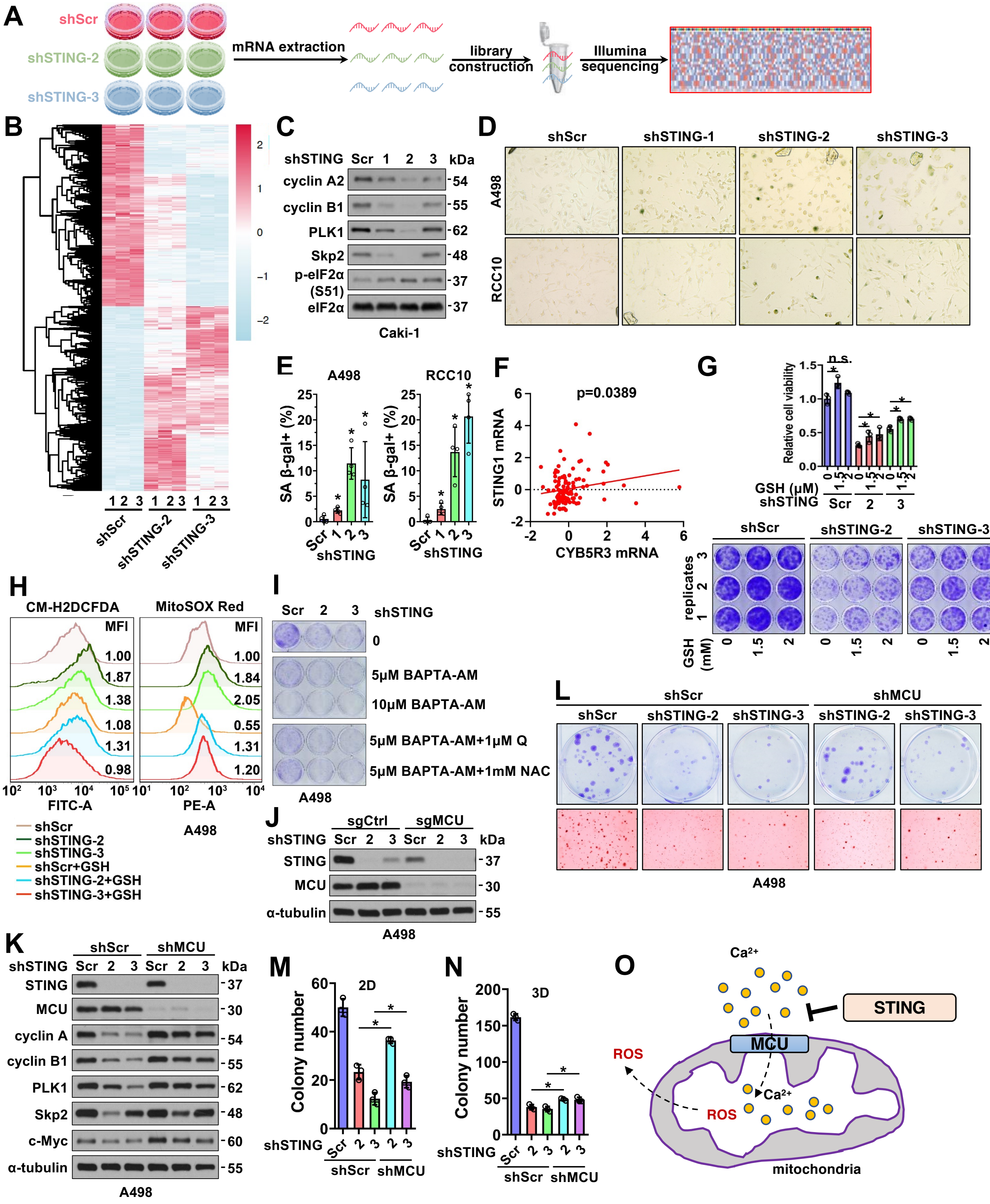
A
shScr
shSTING-2
shSTING-3
mRNA extraction
library construction
Illumina sequencing
B
C
shSTING Scr 1 2 3 kDa
cyclin A2 54
cyclin B1 55
PLK1 62
Skp2 48
p-eIF2α (S51) 37
eIF2α 37
Caki-1
D
shScr shSTING-1 shSTING-2 shSTING-3
A498
RCC10
E
A498
RCC10
SA β-gal+ (%)
shSTING
F
p=0.0389
STING1 mRNA
CYB5R3 mRNA
G
n.s.
Relative cell viability
GSH (μM)
shSTING Scr 2 3
replicates
GSH (mM)
H
CM-H2DCFDA
MitoSOX Red
MFI
FITC-A
PE-A
A498
shScr
shSTING-2
shSTING-3
shScr+GSH
shSTING-2+GSH
shSTING-3+GSH
I
Scr 2 3 shSTING
0
5μM BAPTA-AM
10μM BAPTA-AM
5μM BAPTA-AM+1μM Q
5μM BAPTA-AM+1mM NAC
A498
J
sgCtrl sgMCU
shSTING Scr 2 3 Scr 2 3 kDa
STING 37
MCU 30
α-tubulin 55
A498
K
shScr shMCU
shSTING Scr 2 3 Scr 2 3 kDa
STING 37
MCU 30
cyclin A 54
cyclin B1 55
PLK1 62
Skp2 48
c-Myc 60
α-tubulin 55
A498
L
shScr shMCU
shScr shSTING-2 shSTING-3 shSTING-2 shSTING-3
A498
M
2D
Colony number
shSTING
shScr shMCU
N
3D
Colony number
shSTING
shScr shMCU
O
Ca2+
STING
MCU
ROS
Ca2+
ROS
mitochondria

Figure S3. STING depletion in A498 cells leads to deregulation in cell cycle progression caused by imbalance in mitochondrial calcium homeostasis. (A) A cartoon illustration for the pipeline for RNA-Seq. (B) A heatmap to summarize changes of genes in redox homeostasis (redox gene set in Harmonizome database) in control or STING depleted A498 cells from RNA-Seq. (C) IB analyses of WCL derived from indicated Caki-1 cells. (D) Representative images for β-galactosidase staining using cells from D and quantified in E. Error bars were calculated as mean+/-SD, n=3. *p<0.05 (one-way ANOVA test). (F) mRNA expression of STING1 positively correlates with CYB5R3 analyzed using CPTAC data from [1]. (G) Representative images for 2D colony formation assays using indicated A498 cells treated with indicated doses of GSH. Where indicated, GSH treatment was refreshed every other day for 9 days and quantified by measuring OD590 of resolved crystal violet in 10% acetic acid solution. Error bars were calculated as mean+/-SD, n=3. *p < 0.05 (one-way *ANOVA* test). (H) FACS analyses of cellular ROS levels and mitochondrial ROS levels with indicated treated in indicated A498 cells. (I) Representative images for 2D colony formation assays using indicated A498 cells treated with indicated doses of compounds. Where indicated, BAPTA-AM treatment was refreshed every other day for 7 days. (J, K) IB analyses of WCL derived from indicated A498 cells. (L) Representative images for both 2D colony formation assays and 3D soft agar assays using cells generated in (K) and quantified in (M) and (N). Error bars were calculated as mean+/-SD, n=3. *p < 0.05 (one-way *ANOVA* test). (O) A cartoon illustration showing that STING suppresses calcium transport into mitochondria such that MCU depletion rescues STING depletion induced mitochondria calcium and ROS increases.

A

Enrichment FDR	Genes in list	Total genes	Functional Category
9.8E-78	78	149	Protein localization to ER
5.3E-76	69	111	Selective membrane channel proteins
1.3E-74	67	106	SRP-dependent cotranslational protein targeting to membrane
3.6E-73	70	124	Establishment of protein localization to ER
3.0E-71	68	120	Protein targeting to ER
1.8E-63	78	212	Translational initiation

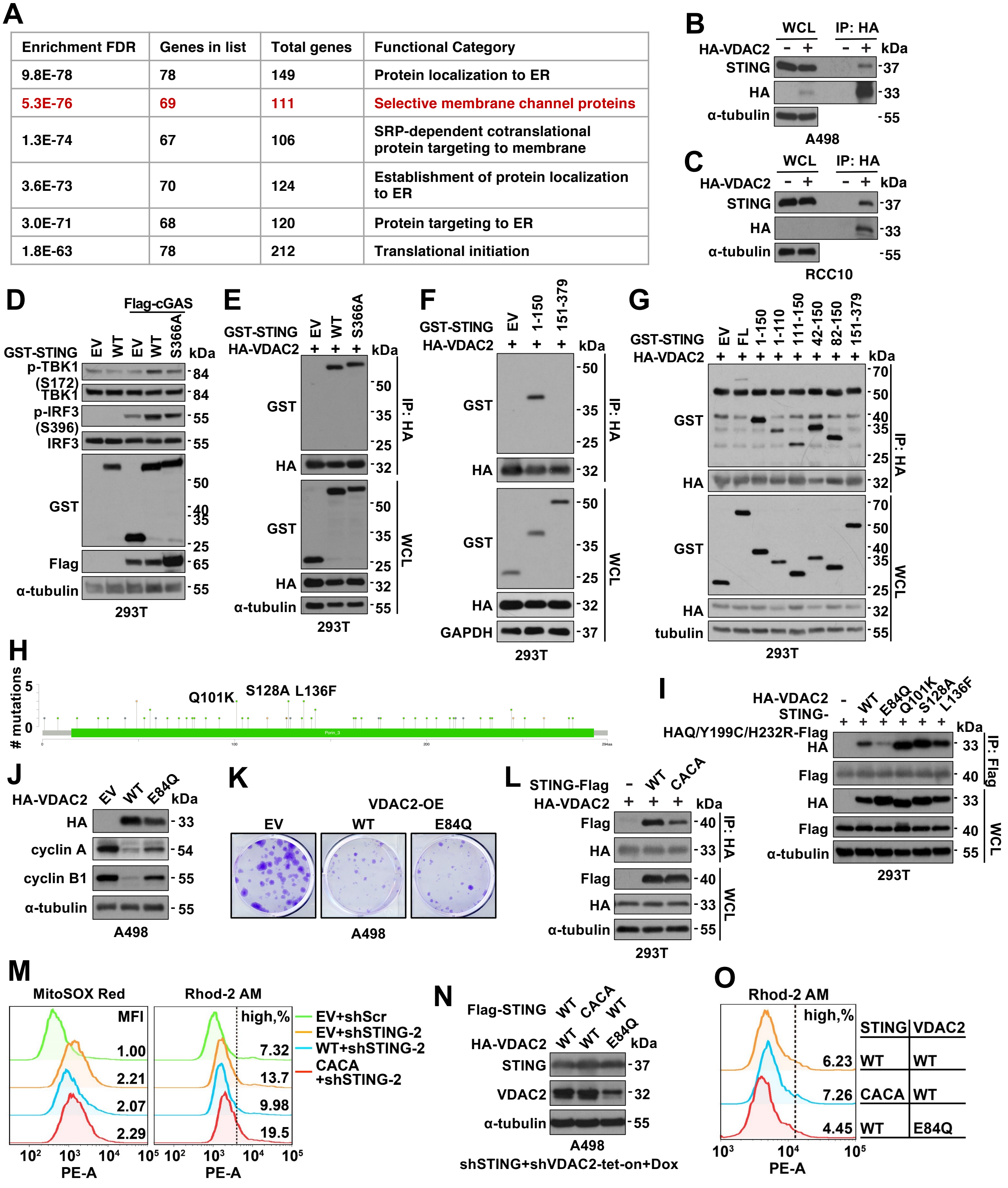

Figure S4. STING binds VDAC2 to regulate mitochondrial calcium homeostasis. (A) Functional analyses of statistically significant STING binding partners. (B, C) IB analyses of HA-IP (right panel) and WCL (left panel) from A498 (B) or RCC10 (C) cells. (D) IB analyses of WCL from HEK293T cells transfected with indicated DNA constructs. (E, F, G) IB analyses of HA-IP and WCL from HEK293T cells transfected with indicated DNA constructs. (H) An illustration of most frequently observed VDAC2 cancer patient derived mutations (from cbioportal). (I) IB analyses of Flag-IP and WCL derived from HEK293T cells transfected with indicated VDAC2 and STING constructs. (J) IB analyses of WCL from A498 cells stably expressing indicated lentiviral HA-VDAC2 constructs. (K) Representative images for 2D colony formation assays using cells obtained from (J). (L) IB analyses of HA-IP and WCL from HEK293T cells transfected with HA-VDAC2 and indicated STING constructs. (M) FACS analyses of mitochondrial ROS (MitoSOX Red) or mitochondrial calcium (Rhod-2 AM) in indicated A498 cells. (N) IB analyses of WCL from iSTING/VDAC2 double depleted A498 cells expressing indicated STING and VDAC2. (O) FACS analyses of mitochondrial calcium (Rhod-2 AM) in indicated A498 cells from N.

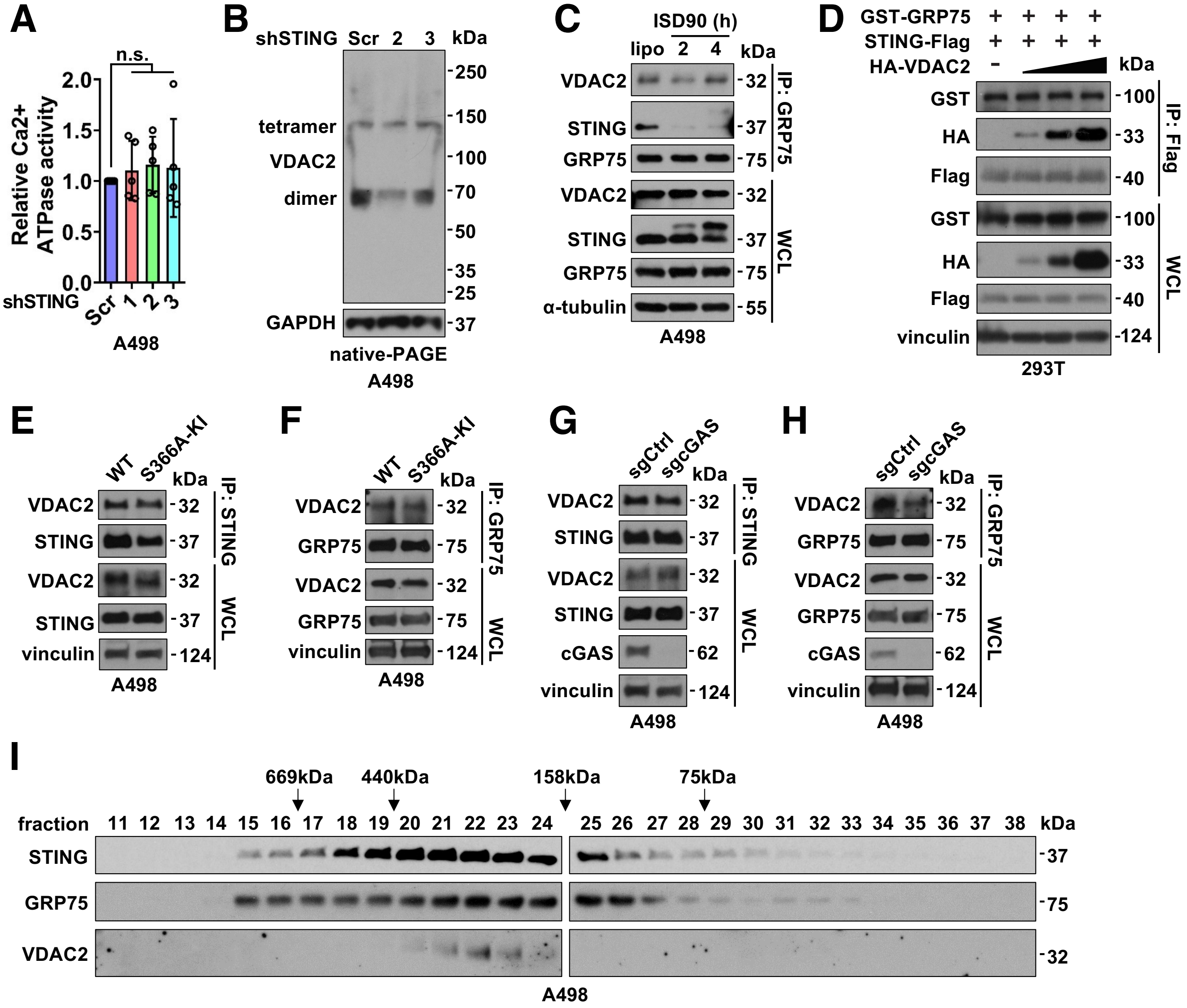

Figure S5. STING depletion does not affect VDAC2 oligomerization but interferes with MERC. (A) A Ca^{2+} related ATPase activity assay using indicated A498 cell lysates. Activity was measured using a Ca^{2+} ATPase activity assay kit (Elabscience) according to manufacturer's protocol. Error bars were calculated as mean+/-SD, n=5. n.s., no significance (one-way *ANOVA* test). (B) IB analyses of WCL in indicated STING depleted A498 cells using native PAGE. (C) IB analyses of GRP75-IP and WCL from A498 cells treated with lipofectamine 3000 or ISD90 (5 μg/mL) for indicated hrs before cell collection. (D) IB analyses of Flag-IP and WCL derived from HEK293T cells transfected with indicated DNA constructs. (E, G) IB analyses of STING-IP and WCL derived from indicated A498 cells. (F, H) IB analyses of GRP75-IP and WCL derived from indicated A498 cells. (IB) IB analyses of FPLC fractions using A498 cell lysates.

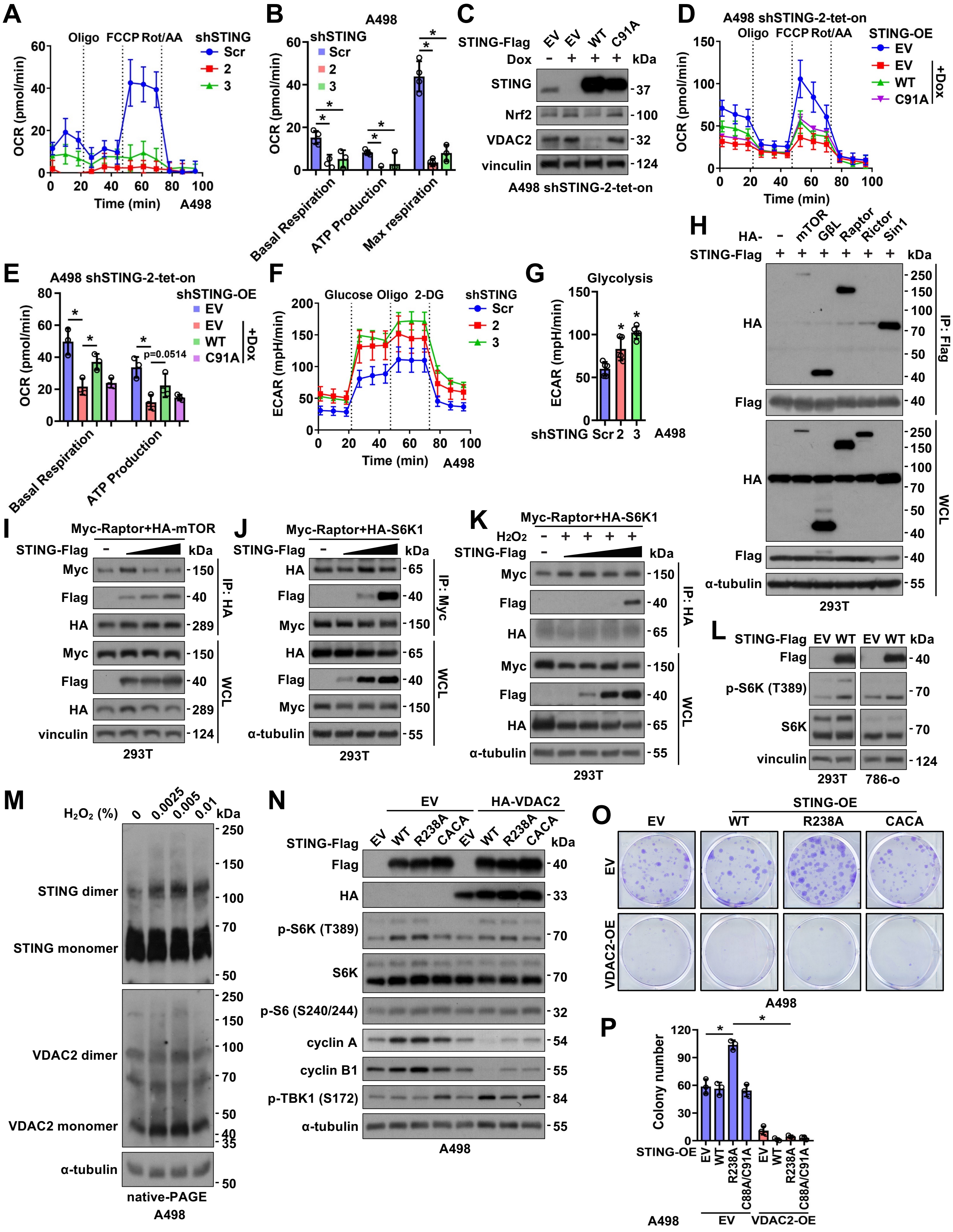
A
Oligo FCCP Rot/AA
shSTING
Scr
2
3
OCR (pmol/min)
Time (min)
A498
B
A498
shSTING
Scr
2
3
OCR (pmol/min)
Basal Respiration
ATP Production
Max respiration
C
STING-Flag EV EV WT C91A
Dox - + + +
kDa
STING
Nrf2
VDAC2
vinculin
A498 shSTING-2-tet-on
D
A498 shSTING-2-tet-on
Oligo FCCP Rot/AA
STING-OE
EV
EV
WT
C91A
+Dox
OCR (pmol/min)
Time (min)
E
A498 shSTING-2-tet-on
shSTING-OE
EV
EV
WT
C91A
+Dox
p=0.0514
OCR (pmol/min)
Basal Respiration
ATP Production
F
Glucose Oligo 2-DG
shSTING
Scr
2
3
ECAR (mpH/min)
Time (min)
A498
G
Glycolysis
ECAR (mpH/min)
shSTING Scr 2 3
A498
H
HA- - mTOR GβL Raptor Rictor Sin1
STING-Flag + + + + + +
kDa
HA
Flag
IP: Flag
WCL
α-tubulin
293T
I
Myc-Raptor+HA-mTOR
STING-Flag
kDa
Myc
Flag
HA
vinculin
IP: HA
WCL
293T
J
Myc-Raptor+HA-S6K1
STING-Flag
kDa
HA
Flag
Myc
α-tubulin
IP: Myc
WCL
293T
K
Myc-Raptor+HA-S6K1
H2O2
STING-Flag
kDa
Myc
Flag
HA
α-tubulin
IP: HA
WCL
293T
L
STING-Flag EV WT EV WT kDa
Flag
p-S6K (T389)
S6K
vinculin
293T 786-o
M
H2O2 (%) 0 0.0025 0.005 0.01 kDa
STING dimer
STING monomer
VDAC2 dimer
VDAC2 monomer
α-tubulin
native-PAGE
A498
N
EV
HA-VDAC2
STING-Flag EV WT R238A CACA EV WT R238A CACA
kDa
Flag
HA
p-S6K (T389)
S6K
p-S6 (S240/244)
cyclin A
cyclin B1
p-TBK1 (S172)
α-tubulin
A498
O
STING-OE
EV WT R238A CACA
EV
VDAC2-OE
A498
P
Colony number
STING-OE EV WT R238A C88A/C91A EV WT R238A C88A/C91A
A498
EV
VDAC2-OE

Figure S6. STING depletion attenuates mitochondria function and suppresses mTORC1/S6K signaling to inhibit RCC cell growth. (A) Normalized OCR plots using indicated shSTING A498 cells. Quantifications are shown in (B). Error bars were calculated as mean+/-SD, n=4. *$p<0.05$ (one-way ANOVA test). (C) IB analysis of WCL from indicated A498-teton-shSTING cells. (D) Normalized OCR plots using shSTING-tet-on A498 cells treated with or without 1 mg/mL doxycycline treatment for 4 days. Quantifications are shown in (E). Error bars were calculated as mean+/-SD, n=3. *$p<0.05$ (one-way ANOVA test) (F) Normalized ECAR plots using indicated shSTING A498 cells. Quantifications are shown in (G). Error bars were calculated as mean+/-SD, n=5. *$p<0.05$ (one-way ANOVA test). (H) IB analyses of Flag-IP and WCL derived from HEK293T cells transfected with indicated HA constructs with Flag-STING. (I) IB analyses of HA-IP and WCL derived from HEK293T cells transfected with indicated doses of Flag-STING with Myc-Raptor and HA-mTOR. (J) IB analyses of Myc-IP and WCL derived from HEK293T cells transfected with indicated doses of Flag-STING with Myc-Raptor and HA-S6K1. (K) IB analyses of HA-IP and WCL derived from HEK293T cells transfected with indicated doses of Flag-STING with Myc-Raptor and HA-S6K1 treated with 0.01% H_2O_2 for 6 hrs before cell collection. (L) IB analyses of WCL from HEK293T cells transfected with indicated DNA constructs or 786-o cells infected with indicated lenti-viruses. (M) IB analyses of WCL from A498 cells treated with indicated doses of H_2O_2. (N) IB analyses of WCL from A498 cells stably expressing indicated STING-Flag and HA-VDAC2 by lentiviral infection. (O) Representative of 2D colony formation images for cells obtained in (M) and quantified in (P). Error bars were calculated as mean+/-SD, n=3. *$p<0.05$ (one-way ANOVA test).

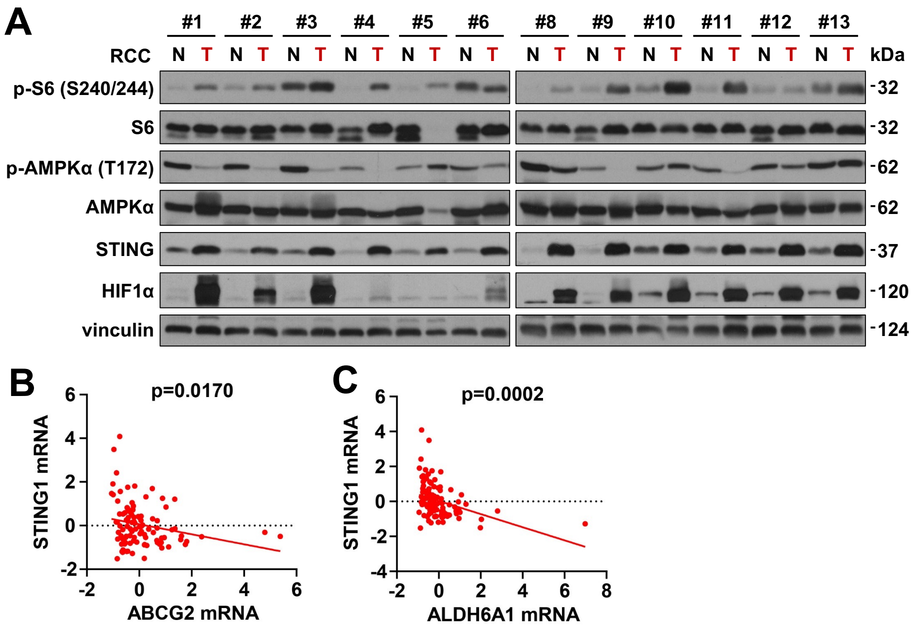

Figure S7. Increased mTORC1 signaling was observed in RCC patient tissues. (A) IB analyses of WCL from 12 pairs of RCC tumor tissues with matched normal adjacent tissues. (B, C) STING mRNA is inversely correlated with expression of NRF2 targets ABCG2 and ALDH6A1 in CPTAC data from [1].

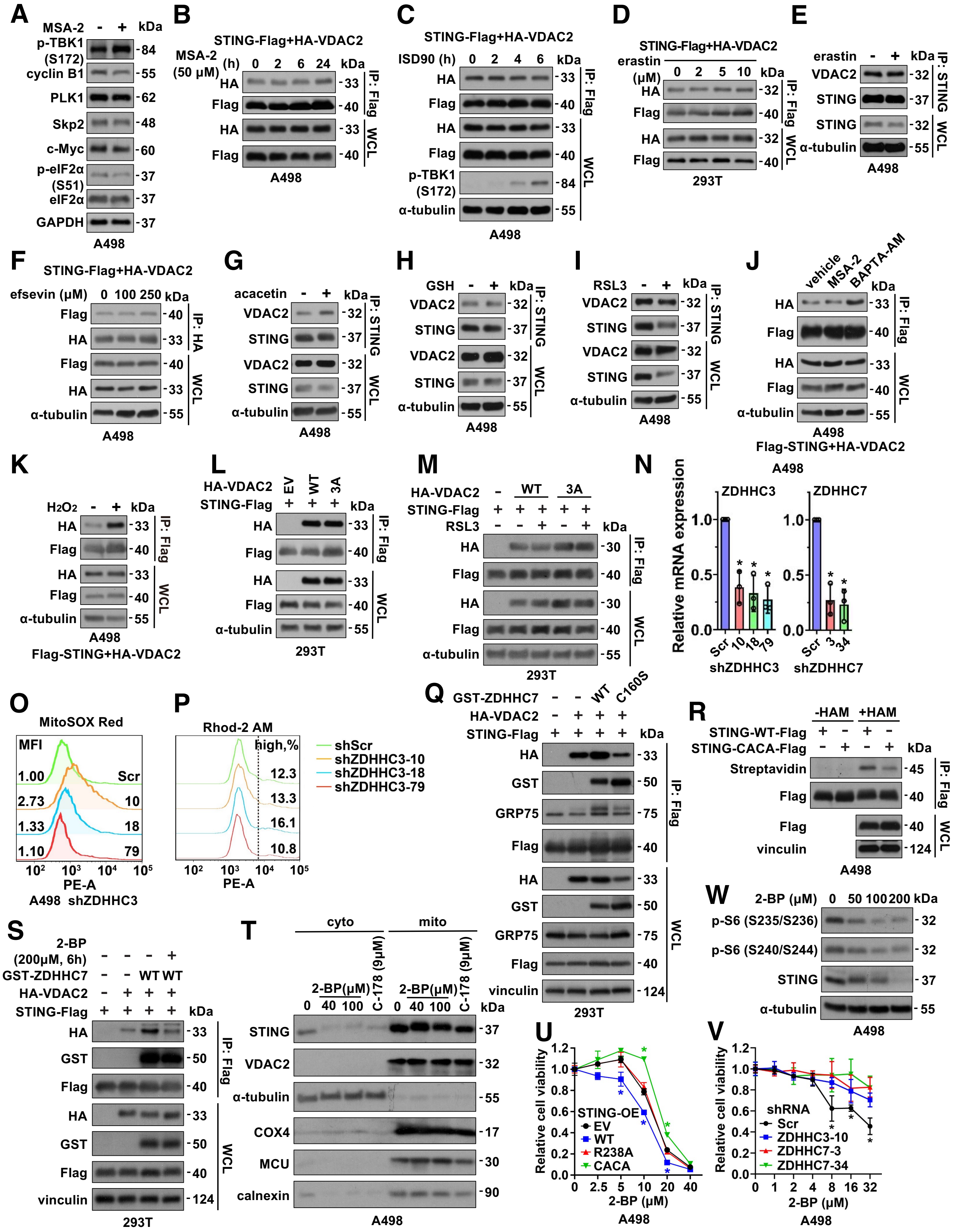
A
MSA-2
kDa
p-TBK1 (S172)
cyclin B1
PLK1
Skp2
c-Myc
p-eIF2α (S51)
eIF2α
GAPDH
A498
B
STING-Flag+HA-VDAC2
MSA-2 (50 μM)
IP: Flag
WCL
C
STING-Flag+HA-VDAC2
ISD90 (h)
D
STING-Flag+HA-VDAC2
erastin (μM)
293T
E
erastin
IP: STING
F
STING-Flag+HA-VDAC2
efsevin (μM)
IP: HA
G
acacetin
H
GSH
I
RSL3
J
vehicle
MSA-2
BAPTA-AM
Flag-STING+HA-VDAC2
K
H2O2
Flag-STING+HA-VDAC2
L
HA-VDAC2
STING-Flag
M
N
Relative mRNA expression
ZDHHC3
ZDHHC7
shZDHHC3
shZDHHC7
O
MitoSOX Red
MFI
PE-A
A498 shZDHHC3
P
Rhod-2 AM
high,%
shScr
shZDHHC3-10
shZDHHC3-18
shZDHHC3-79
Q
GST-ZDHHC7
C160S
GRP75
vinculin
R
-HAM
+HAM
STING-WT-Flag
STING-CACA-Flag
Streptavidin
S
2-BP (200μM, 6h)
GST-ZDHHC7
T
cyto
mito
2-BP(μM)
C-178 (9μM)
COX4
MCU
calnexin
U
Relative cell viability
STING-OE
EV
WT
R238A
CACA
2-BP (μM)
V
shRNA
Scr
ZDHHC3-10
ZDHHC7-3
ZDHHC7-34
W
2-BP (μM)
p-S6 (S235/S236)
p-S6 (S240/S244)

Figure S8. Disrupting STING binding to VDAC2 reduces RCC growth. (A) IB analyses of WCL from A498 cells treated with or without 50 μM MSA-2 for 24 hrs before cell collection. (B, C) IB analyses of Flag-IP and WCL from A498 cells stably expressing Flag-STING and HA-VDAC2 by lentiviral infection treated with 50 μM MSA-2 (B) or 5 μg/ml ISD90 (C) for indicated time periods. (D) IB analyses of Flag-IP and WCL from HEK293T cells transfected with Flag-STING and HA-VDAC2 treated with indicated doses of erastin for 24 hrs prior to cell collection. (E) IB analyses of STING-IP and WCL from A498 cells treated with 5 μM erastin for 24 hrs prior to cell collection. (F) IB analyses of HA-IP and WCL from A498 cells stably expressing Flag-STING and HA-VDAC2 treated with indicated doses of efsevin for 24 hrs prior to cell collection. (G, H, I) IB analyses of STING-IP and WCL from A498 cells treated with 1 μM acacetin (G), 6 mM GSH (H), 0.5 μM RSL3 (I) for 24 hrs prior to cell collection. (J) IB analyses of Flag-IP and WCL from A498 cells stably expressing Flag-STING and HA-VDAC2 by lentiviral infection treated with 50 μM MSA-2 or 10 μM BAPTA-AM for 24 hrs prior to cell collection. (K) IB analyses of Flag-IP and WCL from A498 cells stably expressing Flag-STING and HA-VDAC2 by lentiviral infection treated with 0.1% H_2O_2 for 1 hr prior to cell collection. (L) IB analyses of Flag-IP and WCL derived from HEK293T cells transfected with indicated DNA constructs. (M) IB analyses of Flag-IP and WCL derived from HEK293T cells transfected with indicated DNA constructs and treated with 0.5 μM RSL3 for 3 hrs prior to cell collection. (N) RT-PCR analyses of indicated ZDHHC mRNAs to examine indicated ZDHHC knockdown efficiency. Error bars were calculated as mean+/-SD, n=3. *p<0.05 (one-way ANOVA test). (O) FACS analyses of mitochondria ROS (MitoSOX Red) in indicated A498 cells. (P) FACS analyses of mitochondria calcium (Rhod-2 AM) in indicated A498 cells. (Q) IB analyses of Flag-IP and WCL derived from HEK293T cells transfected with indicated DNA constructs. (R) IB analyses of Flag-IP and WCL derived from A498 cells overexpressing STING-WT and -CACA. (S) IB analysis of indicated fractions from A498 cells treated with indicated compounds for 24 hrs. (T) IB analysis of WCL from A498 cells treated with indicated doses of 2-BP overnight before cell collection. (U, V) Analyses of relative viability of indicated A498 cells treated with indicated doses of 2-BP for 4 days before cell viability is measured. Error bars were calculated as mean+/-SD, n=3.

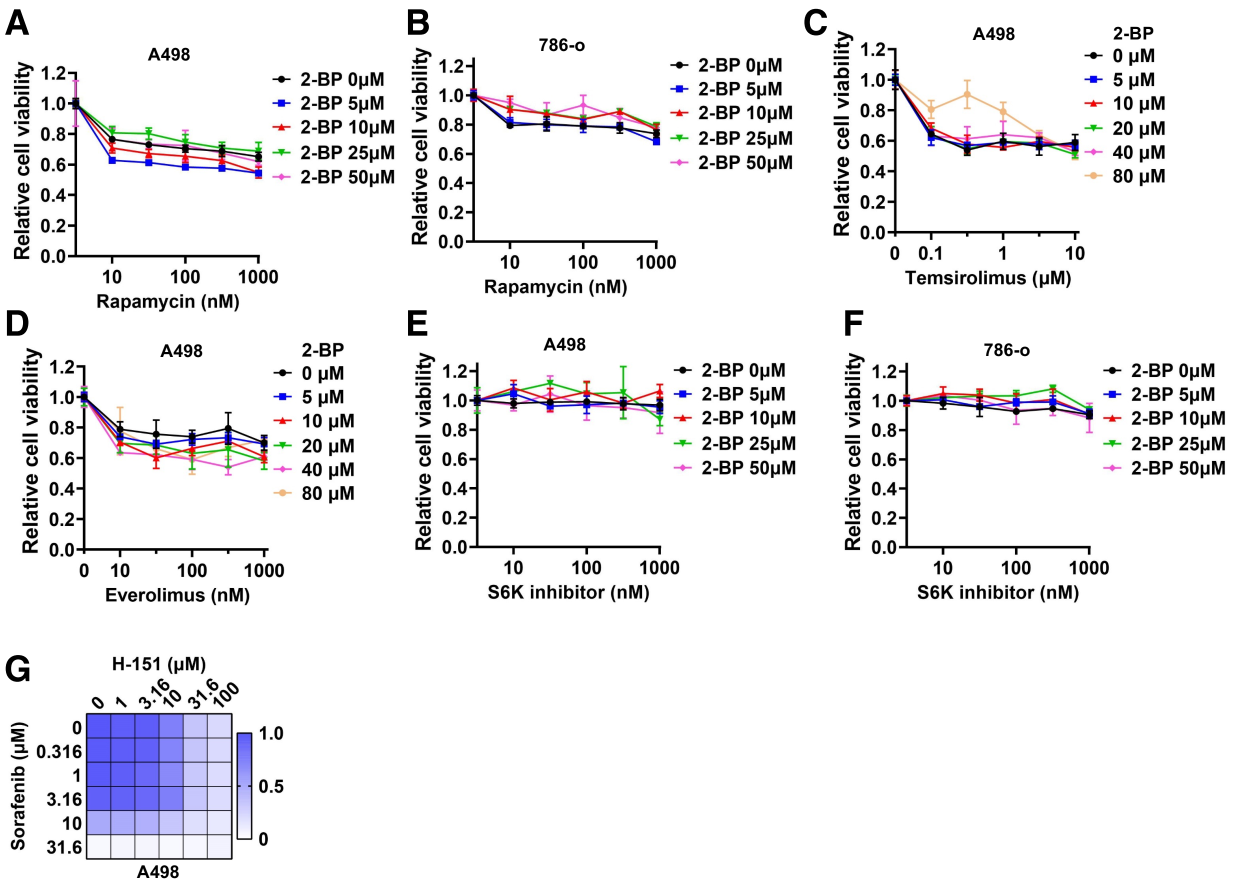

Figure S9. 2-BP and sorafenib displays a synergy in suppressing A498 tumor growth. (A-F) Analyses of relative viability of indicated RCC cells treated with indicated doses of 2-BP and indicated compounds for 2 days before cell viability is measured. Error bars were calculated as mean+/-SD, n=3. (G) A representative heatmap for viability of A498 cells treated with indicated doses of compounds for 4 days.

References:

1. Clark, D.J., Dhanasekaran, S.M., Petralia, F., Pan, J., Song, X., Hu, Y., da Veiga Leprevost, F., Reva, B., Lih, T.M., Chang, H.Y., *et al.* (2019). Integrated Proteogenomic Characterization of Clear Cell Renal Cell Carcinoma. Cell *179*, 964-983 e931.
